# The State of the Art of eHealth Self-Management Interventions for People With Chronic Obstructive Pulmonary Disease: Scoping Review

**DOI:** 10.2196/57649

**Published:** 2025-03-10

**Authors:** Eline te Braake, Roswita Vaseur, Christiane Grünloh, Monique Tabak

**Affiliations:** 1 Roessingh Research and Development Enschede The Netherlands; 2 Faculty of Electrical Engineering, Mathematics, and Computer Science Biomedical Signals and Systems Group University of Twente Enschede The Netherlands

**Keywords:** eHealth, self-management, interventions, chronic obstructive pulmonary disease, COPD, review

## Abstract

**Background:**

Chronic obstructive pulmonary disease (COPD) is a common chronic incurable disease. Treatment of COPD often focuses on symptom management and progression prevention using pharmacological and nonpharmacological therapies (eg, medication, inhaler use, and smoking cessation). Self-management is an important aspect of managing COPD. Self-management interventions are increasingly delivered through eHealth, which may help people with COPD engage in self-management. However, little is known about the actual content of these eHealth interventions.

**Objective:**

This literature review aimed to investigate the state-of-the-art eHealth self-management technologies for COPD. More specifically, we aimed to investigate the functionality, modality, technology readiness level, underlying theories of the technology, the positive health dimensions addressed, the target population characteristics (ie, the intended population, the included population, and the actual population), the self-management processes, and behavior change techniques.

**Methods:**

A scoping review was performed to answer the proposed research questions. The databases PubMed, Scopus, PsycINFO (via EBSCO), and Wiley were searched for relevant articles. We identified articles published between January 1, 2012, and June 1, 2022, that described eHealth self-management interventions for COPD. Identified articles were screened for eligibility using the web-based software Rayyan.ai. Eligible articles were identified, assessed, and categorized by the reviewers, either directly or through a combination of methods, using Atlas.ti version 9.1.7.0. Thereafter, data were charted accordingly and presented with the purpose of giving an overview of currently available literature while highlighting existing gaps.

**Results:**

A total of 101 eligible articles were included. This review found that most eHealth technologies (91/101, 90.1%) enable patients to self-monitor their symptoms using (smart) measuring devices (39/91, 43%), smartphones (27/91, 30%), or tablets (25/91, 27%). The self-management process of “taking ownership of health needs” (94/101, 93.1%), the behavior change technique of “feedback and monitoring” (88/101, 87%), and the positive health dimension of “bodily functioning” (101/101, 100%) were most often addressed. The inclusion criteria of studies and the actual populations reached show that a subset of people with COPD participate in eHealth studies.

**Conclusions:**

The current body of literature related to eHealth interventions has a strong tendency toward managing the physical aspect of COPD self-management. The necessity to specify inclusion criteria to control variables, combined with the practical challenges of recruiting diverse participants, leads to people with COPD being included in eHealth studies that only represent a subgroup of the whole population. Therefore, future research should be aware of this unintentional blind spot, make efforts to reach the underrepresented population, and address multiple dimensions of the positive health paradigm.

## Introduction

### Background

Chronic obstructive pulmonary disease (COPD) is a common disabling lung condition characterized by chronic respiratory symptoms that cause persistent, mostly progressive airflow limitations [1]. It is one of the major issues of public health, and its prevalence, mortality, and morbidity are increasing [1-3]. COPD was listed as the third leading cause of death worldwide in 2019 [4], and it is estimated that by 2040, deaths from COPD will rise to 4.4 million per year [5]. In addition, people with a lower socioeconomic status are at increased risk of developing COPD [6]. Although COPD may, in some cases, be the result of a genetic risk factor, it is, in most cases, caused by exposure to tobacco smoking and the inhalation of toxic particles and gases from indoor and outdoor air pollution [7,8]. People with COPD often experience symptoms such as dyspnea, fatigue, chest tightness, activity limitation, and cough that may be accompanied by sputum production [7]. Furthermore, they may experience acute events, known as exacerbations, which can lead to hospitalization. Although COPD is chronic and thus not curable, it is, however, treatable, and disease progression is preventable [3,9]. Therefore, the treatment of COPD often focuses on reducing symptoms and future risks of exacerbation with the use of pharmacological and nonpharmacological therapies (eg, inhaler use, vaccinations, smoking cessation, and self-management) [7]. Given its chronic nature and the impact of the disease on all facets of one’s life, an important aspect of treating COPD and secondary prevention is chronic disease management.

An essential component of chronic disease management is self-management [10]. Owing to the variation in the literature regarding the definition of self-management in COPD, this paper defines self-management as follows: “The ability of an individual to manage one’s symptoms, treatment, physical, social, and emotional consequences, and lifestyle changes. It includes means of empowerment, educating oneself, being autonomous, learning and adapting to new behaviors, acceptance, and adapting to a new balance in life.” It requires patients to take an involved and responsible role in their health, with the aim of becoming active participants [10,11]. Self-management interventions or programs are developed to help patients engage in self-management, and their effectiveness is investigated in research.

Self-management interventions or programs are shown to have positive effects, for example, in supporting patients to develop and improve their self-management skills and disease knowledge [12-14]. Camus-García et al [15] found that self-management interventions may improve clinical outcomes in COPD (eg, improvements in health-related quality of life) and lower the probability of hospital admissions. The actual content of such self-management intervention programs for COPD is diverse [12], and it remains unclear which specific elements are essential for designing a successful program. In the following sections, some elements that can be considered when designing an intervention program will be briefly described: content for COPD self-management, processes of self-management, and behavior change techniques (BCTs).

The diversity of content may be explained by the numerous objectives and end points of self-management intervention programs [16]. Interventions focus on acute exacerbation management and admission avoidance by incorporating exacerbation action plans [17] and often also include education, exercise training, and breathing strategies [18]. However, research suggests that intervention programs that only include education or action plans alone may not result in behavioral change, increased patient confidence, or the acquisition of new skills that patients learn or practice [16,19].

Besides the content of self-management intervention programs, the design of the intervention program should also reflect that self-management consists of different processes. Schulman-Green et al [20] identified different self-management processes for chronic illnesses, such as “learning,’” “taking ownership of health needs,” and “performing health promotion activities.” All processes are divided into specific self-management tasks (eg, “learning about condition and health needs” and “changing behavior to minimize health impact”) and skills (eg, “acquiring information” and “reducing stress”) [20]. Schulman-Green et al [20] concluded that the identification of such processes may help support and guide future self-management intervention programs. They also demonstrated that these various processes should be viewed within the broader context, as their significance to patients may vary depending on where they are in their patient journey [20]. Therefore, more knowledge about such self-management processes within self-management eHealth intervention programs is needed to support the development of such interventions.

Self-management interventions may also aim to change a certain behavior of the patient, so the incorporation of BCTs can be beneficial to designing a successful intervention program. A BCT is “a specific observable, replicable, and irreducible component of an intervention program designed to alter or redirect causal processes that regulate behavior” [21] and can be included in the design of any type of self-management intervention program. By adding these “active ingredients” (eg, “feedback” and “self-monitoring”), chances for achieving behavioral change may be increased [21]. Thus, combining self-management processes and BCTs in intervention programs may lead to positive results for one’s self-management. However, to the best of our knowledge, no research is dedicated to investigating the presence of BCTs and self-management processes in current self-management interventions for COPD.

One way to support people with COPD in engaging in self-management is through the use of eHealth interventions. eHealth interventions can be defined as “An eHealth technology specifically focused on intervening in an existing context by changing behaviors and/or cognitions” [22]. eHealth interventions to support self-management may help people with chronic diseases become more independent and empowered by, for example, gaining knowledge about their disease, monitoring and reporting daily symptoms, and learning specific self-management skills [23-25]. Therefore, the use of eHealth interventions in COPD care represents a promising way of delivering health services, such as support in self-management [26].

In the current literature, a diverse range of eHealth interventions aim to support patients in their self-management, and these are increasingly provided to support patients in health communication, self-monitoring, and their medical treatment [10,26]. Available literature revealed that current eHealth interventions for COPD mainly focused on COPD care, education, smoking cessation, medication adherence, exercise, diet, and symptom management [27,28]. This indicates a tendency toward managing the physical aspect of COPD in self-management eHealth interventions. However, the physical aspect of one’s disease is only 1 dimension of the positive health paradigm. As conceptualized by Huber et al [29], “Health includes the ability to adapt, and self manage in the face of social, physical, and emotional challenges,” also referred to as “positive health.” Huber et al [30] stated that positive health as a concept has several important health indicators, categorized into 6 dimensions: “bodily functions,” “mental well-being,” “meaningfulness,” “quality of life,” “social participation,” and “daily functioning” [30]. They stressed the fact that paying attention to these indicators could support shared decision-making and bridge the gap between health care and the social context. Therefore, these dimensions are all important to consider when self-managing one’s disease. However, no research is available regarding the extent of positive health dimensions addressed in current self-management eHealth interventions for COPD.

Furthermore, using eHealth to support people with COPD might entail some challenges, as low health literacy is prevalent among people with COPD [31,32]. In addition, moderate levels of self-reported eHealth literacy are common among people with COPD [33]. Some studies revealed that people with COPD experienced technical barriers when using eHealth interventions for self-management [34]. However, Williams et al [35] indicated a few technical issues experienced by people with COPD when using eHealth to support self-management, leading to uncertainty about whether such eHealth technologies are suitable for the whole COPD population. Although some information about eHealth use for this population is available [27-28,33,35-36], little research is dedicated to investigating whether current eHealth interventions account for the wider population of people with COPD (such as those with eHealth literacy). Therefore, it should be investigated whether there is a difference between the intended population that eHealth interventions aim to target and the eventual included population in those studies. As knowledge and new insights derived from those studies often serve as a starting point for future work, it can be very valuable to look into the representation of the COPD population within studies.

### Objectives

**Figure 1:**
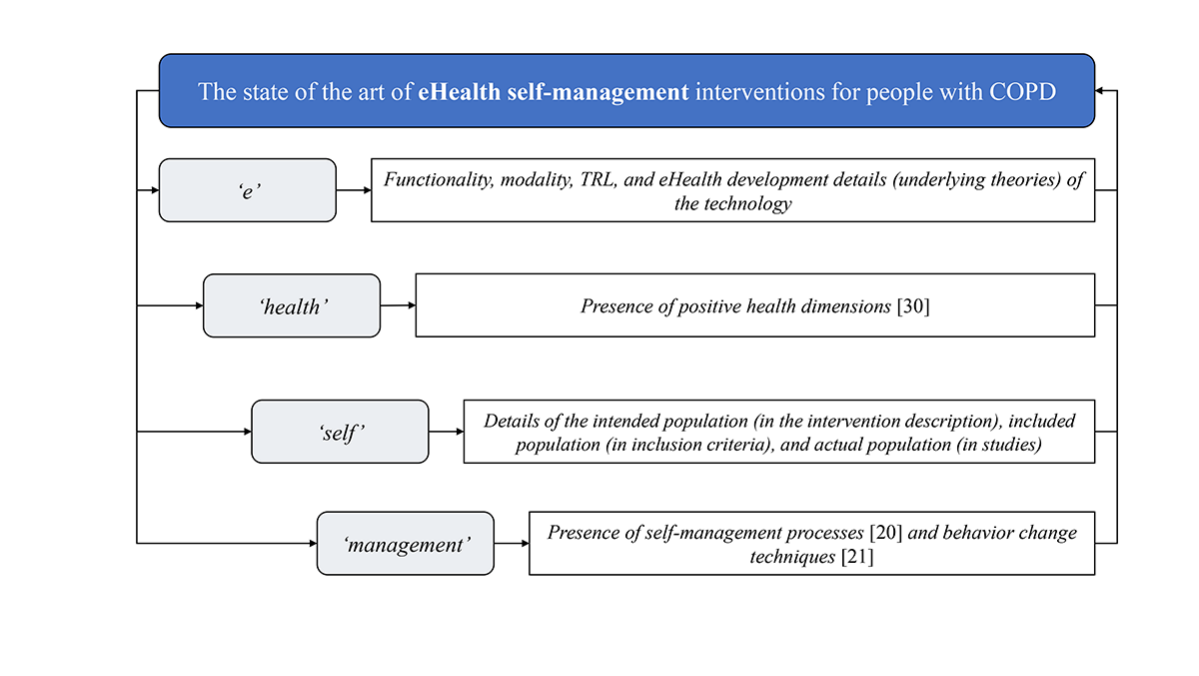
Flowchart of the research questions. COPD: chronic obstructive pulmonary disease; TRL: technology readiness level.

To summarize, little is known about the actual content and design of self-management eHealth interventions for people with COPD. Therefore, this scoping review aimed to investigate the current state-of-the-art eHealth interventions for COPD self-management and identify potential gaps in the literature, which may provide insight into or serve as inspiration for the development of future eHealth self-management interventions. “State of the art” within the context of this study can be defined as follows: “The collection of all underlying components that form the basis for the eHealth self-management interventions for people with COPD.” Figure 1 shows how the different parts of this review contribute to an overall picture of the current literature and highlights the specific aspects explored in this review. More specifically, we aimed to unravel the state of the art of eHealth self-management interventions by using the following subquestions:

- What is the “e” in eHealth self-management?
- What is the “health” in eHealth self-management?
- Who is the “self” in self-management?
- What is the “management” in eHealth self-management?

## Methods

### Overview

A scoping review was performed to investigate the currently available literature. According to Munn et al [37], a scoping review is an ideal tool for providing, for example, an overview of existing literature, identifying key characteristics, and highlighting knowledge gaps, among others. Hence, this was deemed the most suitable method for answering the proposed research questions. Parts of the PRISMA-ScR (Preferred Reporting Items For Systematic Reviews and Meta-Analyses extension for Scoping Reviews) protocol (items 1-7, 9-11, and 13-21), as proposed by Tricco et al [38], were followed and tailored to this study to ensure a systematic approach for answering the research questions. The PRISMA-ScR checklist is provided in Multimedia Appendix 1 [38]. The protocol for this review was not published.

### Search Strategy

The first reviewer (EtB) was responsible for identifying relevant articles in the databases PubMed, Scopus, PsycINFO, and Wiley. Combinations of the search terms “self-management,” “COPD,” and “eHealth” were used to generate the search string. The search string that was used for this review is provided in Multimedia Appendix 2.

### Study Selection

Studies were considered eligible if they were original research and portrayed an eHealth intervention supporting the self-management of COPD. The eHealth self-management intervention should actively involve and engage individuals with COPD, ensuring they experience personal benefits from their self-management efforts, supported and encouraged by the intervention. Per definition, self-management aims for patients to become active participants in their care, which means that patient involvement within self-management eHealth interventions is essential to be able to fulfill an active role. Therefore, we did not consider that eHealth interventions actually support self-management when patients themselves are not involved (eg, if the interaction with the eHealth service is limited to collecting data). Furthermore, articles needed to be published between January 1, 2012, and June 1, 2022. As of 2012, eHealth technologies were upcoming, and their relevance for future health care appeared to be promising. For example, national efforts to implement eHealth in current care were presented in 2012 in the Netherlands (eg, [39]). The complete list of assessment and eligibility criteria is presented in Textbox 1.

**Assessment and eligibility criteria for studies.**

**Concept**

- Studies describing an eHealth intervention supporting the self-management of chronic obstructive pulmonary disease (COPD) were included.
- Studies not fulfilling the inclusion criteria were excluded.

**Population**

- Studies involving adults aged ≥18 years diagnosed with COPD (and other chronic conditions provided that the eHealth technology has a dedicated part toward COPD) were included.
- Studies using general terms such as “older adults,” “rural patients,” or “communities” or referencing unspecified multimorbidity or chronic conditions without any clarification about the population were excluded.

**eHealth technology**

- Studies where eHealth technologies were used to support people with COPD in engaging in self-management, involving patients in their intervention were included if they used at least 1 self-management process, as defined by Schulman-Green et al [20], and in the case of sole monitoring, where patients were able to see their data.
- Studies collecting data solely for research purposes to train machine learning or artificial intelligence algorithms without any further patient engagement were excluded.

**Study design**

- Original research studies were included.
- Reviews, protocols, abstracts, letters, conference proceedings, commentaries, notes, short surveys, and erratum were excluded.

**Language**

- Studies in English were included.
- Studies not fulfilling the inclusion criteria were excluded.

**Year of publication**

- Studies published between January 1, 2012, and June 1, 2022, were included.
- Studies not fulfilling the inclusion criteria were excluded.

### Procedure

The screening was performed on June 3, 2022, with screening supported by the web-based software Rayyan.ai (Rayyan Systems Inc) [40]. To screen articles for title and abstract, both reviewers (EtB and RV) adhered to the eligibility criteria that were discussed before the start of the screening (Textbox 1). One reviewer (EtB) screened all articles for title and abstract. The second reviewer (RV) screened 20% (118/588) of the titles and abstracts of those studies. After this first screening, a discussion took place to compare discrepancies and come to a consensus between reviewers (EtB and RV). Both reviewers had previous experience with performing a systematic review. After this first screening, it was necessary to revise and clarify some of the inclusion criteria to arrive at a satisfactory level of agreement between reviewers. This means that with the use of the revised inclusion criteria, clear and substantiated decisions could be made on whether to include a certain article in this review. For the full-text screening, the same process was applied. In this screening, the level of agreement between reviewers was satisfactory. Reasons for excluding articles during the full-text screening were recorded. Before extracting the data, a data extraction form was developed, discussed, and agreed upon with 3 authors (EtB, CG, and MT). This form was piloted after the full-text screening to reduce errors during data extraction. Data extraction was performed by the first author (EtB) using Atlas.ti (version 9.1.7.0; Lumivero) [41], based on the data extraction form. Some of the data to answer the subquestions were directly extracted (eg, type of study and year of study), some data were the result of an assessment or categorization of the reviewers (eg, positive health dimensions), and some data were a combination of both (eg, BCTs). After the extraction, data were clustered and charted in various ways (eg, bar charts, tables, and descriptive presentations). Finally, charted data were scrutinized and synthesized by 1 reviewer (EtB) before discussing the results with 2 authors (CG and MT). Thereafter, results were written down to answer the proposed research questions. An overview of how the articles were extracted and charted is provided in Multimedia Appendix 3.

## Results

### Search Results

**Figure 2:**
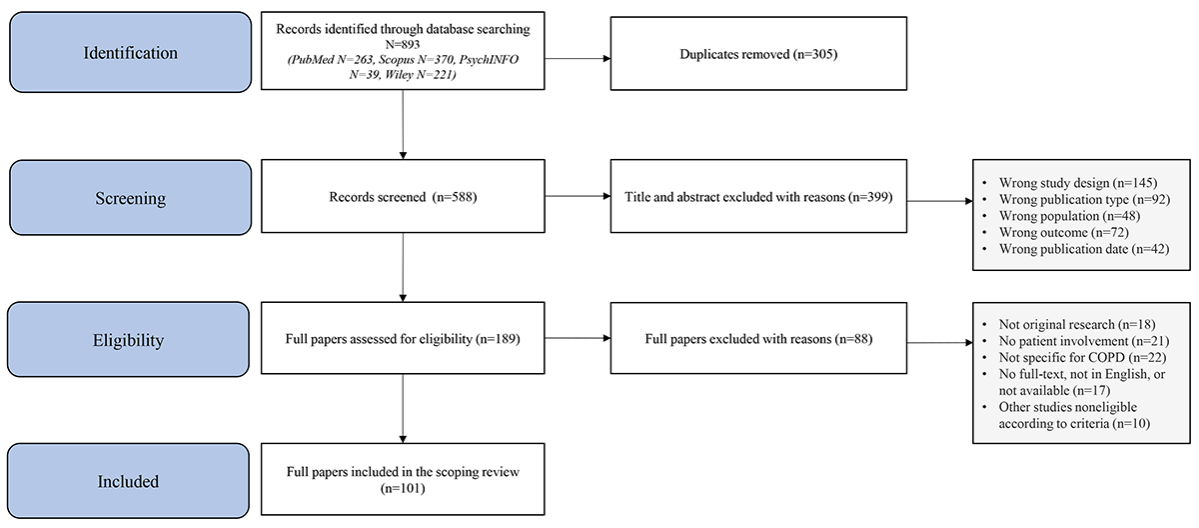
Flowchart of the inclusion of studies. COPD: chronic obstructive pulmonary disease.

Figure 2 shows the detailed flowchart of the inclusion of studies. A total of 893 articles were identified during the initial search, of which 305 (35.1%) duplicates were removed, and the remaining 588 (%) articles were screened on title and abstract; this screening phase resulted in 189 (32.1%) articles that could be assessed for full text. After full-text screening of 189 articles, 88 articles (46.6%) were excluded, resulting in 101 articles (53.4%) being included in this scoping review.

### Study Characteristics

The included papers represented 101 unique studies. Most articles (18/101, 17.8%) were published in 2021, followed by 2020 (15/101, 14.8%) and 2017 (13/101, 12.9%). As shown in Table 1, the most common study types were either randomized controlled trials (18/101, 17.8%) or (prospective) pilot studies (16/101, 15.8%).

**Table 1:** Type of studies (n=101).

Type of studies	Frequency, n (%)
Randomized controlled trials	18 (17.8)
Prospective pilot study	17 (16.8)
Evaluation study	10 (9.9)
Feasibility study	9 (8.9)
Qualitative study	9 (8.9)
Retrospective or secondary analysis	7 (6.9)
Observational study	6 (5.9)
Design or development study	4 (4)
Implementation study	3 (3)
Mixed methods study	3 (3)
Exploratory study	3 (3)
Others	12 (11.9)

### The e in eHealth

This section focuses on the functionality, modality, technology readiness level (TRL) [42], and eHealth development details of the used technologies. Details about the functionality and modality of the eHealth interventions are provided in Multimedia Appendix 4 [25-27,32,36,43-139]. Of the 101 included studies, 76 (75.2%) mentioned the name of their eHealth technologies. Some articles reported on studies using the same eHealth technologies (eg, “EDGE” [35,51-54], “It’s Life!” [43,55,56], “MasterYourBreath” [57-60], and “COMET” [61,62]). In some cases, studies using the same eHealth technology together portrayed the process of developing, testing, and evaluating an eHealth intervention. A total of 50 unique eHealth technologies were found in this review.

Most articles (91/101, 90.1%) included self-monitoring (eg, monitoring of symptoms) as a function of their technology. Of the 101 articles, 69 (68.3%) included the function of educating or informing (eg, education on COPD) and 27 (26.7%) supported communication (eg, eConsults with HCPs and peer-to-peer support chats). Most articles (68/101, 67.3%) included >1 function within their technology.

Table 2 shows that a (smart) measurement device (eg, wearable or monitoring system) was the most common (39/101, 38.6%) modality used in the studies, followed by a smartphone (27/101, 26.7%) and tablet (25/101, 24.7%). If studies used >1 device, the most common combination was a (smart) measurement device with a tablet (19/101, 18.8%) or smartphone (8/101, 7.9%).

**Table 2:** Overview of modalities of the eHealth technologies. Some articles had used >1 device, resulting in 131 modalities within 101 articles.

Modalities of the eHealth technology	Frequency, n (%)
Smart measurement and monitoring devices	45 (34.4)
Smartphone	26 (19.8)
Normal phone or landline	16 (12.2)
Computer, laptop, or whiteboard	16 (12.2)
Tablet	15 (11.5)
Not mentioned	9 (6.9)
Television	3 (2.3)
Other	1 (0.8)

This review found no article that explicitly stated their TRL. According to our assessment and categorization, 47 eHealth technologies in the articles were assessed to be in the development phase (TRL 4 to TRL 6), 53 in the deployment phase (TRL 7 to TRL 9), and 0 in the research phase (TRL 1 to TRL 3).

Details about the eHealth development process showed that only 14 (13.9%) out of 101 studies explicitly mentioned using either a user-centered design, participatory design, scenario-based methods, reflective lifeworld research, or action research approach. Furthermore, of the 101 studies, 18 (17.8%) reported details about the theories on which their self-management intervention was based. Some of these were targeted toward BCTs independent of technology use, while others were technology related and more targeted toward technological adoption or persuasive design. Table 3 lists the various theories identified as underlying the eHealth interventions. Theories that were present could be divided into the following categories: behavioral change, technological adoption or persuasive design, and unspecified. This review found 11 different behavior change theories, 3 different technological adoption or persuasive design theories, and 3 unspecified theories. Of all the different theories within the different categories, the social cognitive theory was most often used (5/11, 45%).

**Table 3:** Theories used within the eHealth self-management interventions.

Categories and theories		References
**Behavioral change**		
	HBM^a^	[60,63]
	Social cognitive theory	[60,64-67]
	Self-care theory	[68]
	Transtheoretical model	[60,64]
	Five A’s model	[56]
	ASE^b^	[60]
	Self-efficacy theory	[69]
	I-Change model	[57-60]
	Self-determination model	[70]
	TGG^c^	[71]
	Theory of planned behavior	[60]
**Technological adoption or persuasive design**		
	TAM^d^	[72]
	UTAUT^e^	[73]
	eHealth-based PCC^f^	[74]
**Unspecified**		
	Goal setting theories	[60]
	Implementation theory	[60]
	Health promotion	[69]

^a^HBM: health belief model.

^b^ASE: attitude-social influence-self-efficacy model.

^c^TGG: Tech to Goal.

^d^TAM: technology acceptance model.

^e^UTAUT: unified theory of acceptance and use of technology.

^f^PCC: person-centered care.

### The Health in eHealth Technologies for Self-Management

Table 4 shows how many eHealth technologies used in the studies addressed the different positive health dimensions. All the included articles (N=101, 100%) addressed (at least) the dimension of bodily functioning, 45 (44.6%) addressed daily functioning, 13 (12.9%) addressed participation, and 12 (11.9%) addressed mental well-being. We were not able to identify any indications that the dimensions of meaningfulness and quality of life were explicitly addressed in any of the eHealth technologies supporting self-management. Details about the positive health dimensions are provided in Multimedia Appendix 5.

Most studies (48/101, 47.5%) focused on 1 specific dimension, namely, bodily functioning. Other articles (42/101, 41.6%) focused on 2 dimensions, 11 (10.9%) on 3 dimensions, and only 3 (3%) on 4 dimensions within their eHealth technology. The combination of the dimensions of bodily functioning and daily functioning was the most common (33/101, 32.7%), followed by the combinations of bodily functioning, daily functioning, and mental well-being (5/101, 5%); bodily functioning and participation (4/101, 4%); bodily functioning, daily functioning, and participation (3/101, 3%); bodily functioning, mental well-being, and participation (3/101, 3%); body functioning, mental well-being, participation, and daily functioning (3/101, 3%); and bodily functioning and mental well-being (1/101, 1%).

**Table 4:** Distribution of the positive health dimensions. Some technologies addressed >1 dimension, which are counted separately in this table, resulting in 172 dimensions found within 101 articles.

Positive health dimension	Frequency, n (%)
Bodily functioning	101 (58.7)
Daily functioning	46 (26.7)
Participation	13 (7.6)
Mental well-being	12 (7)
Meaningfulness	0 (0)
Quality of life	0 (0)

**Figure 3:**
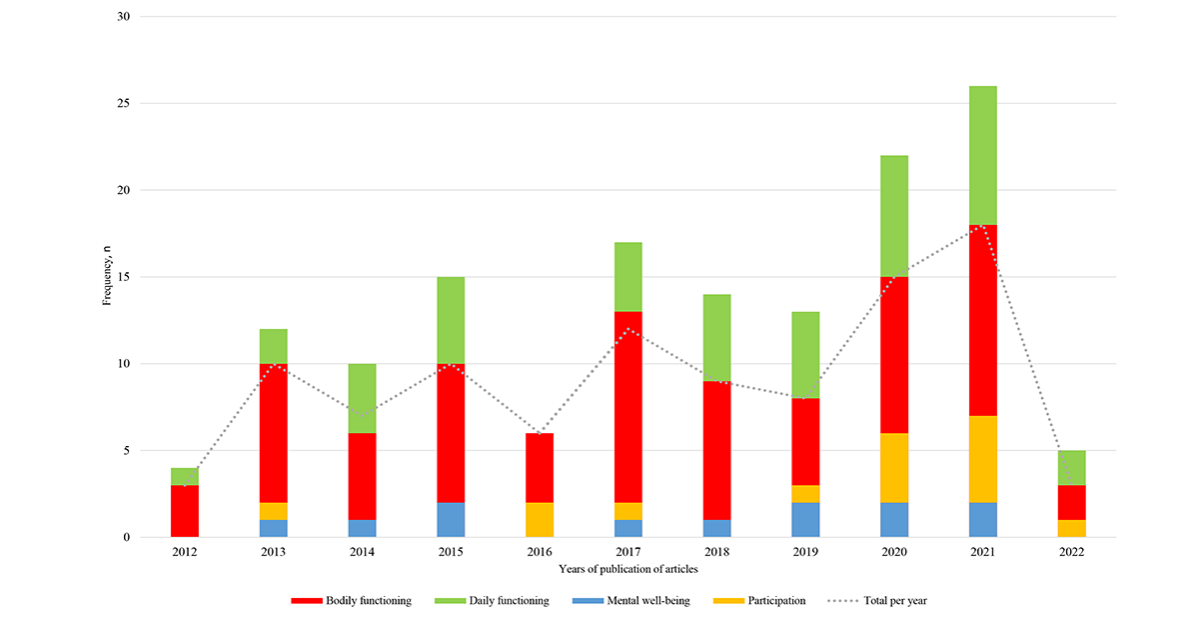
Distribution of the positive health dimensions in articles over time. Dimensions of “quality of life” and “meaningfulness” were not displayed, as no included articles explicitly addressed these.

To investigate whether there may be an increase or decrease in certain positive health dimensions over time, we compared the presence of certain dimensions with the years of the studies (Figure 3). Such information can be useful to see whether the target dimensions of eHealth interventions are changing with time. When comparing the presence of the dimensions with the years of the studies, we found that in the years 2013 to 2015 and 2017 to 2018, the dimension of bodily functioning is dominantly present, followed by daily functioning. From 2017 to 2021, a small increase in the presence of the dimension of mental well-being could be seen over the years. In the years 2020 and 2021, the presence of the dimensions of daily functioning and participation was almost equal compared to bodily functioning.

### The Self in Self-Management

#### Overview

All 101 included papers (partly) described the intended population for the intervention (as stated in the intervention description), the included population (as stated in the inclusion and exclusion criteria), and the final actual study population (as stated in the demographics of study participants). In some studies, certain inclusion criteria were required to participate, thereby restricting the group of eligible participants (ie, the actual population). This scoping review extracted the following inclusion criteria: disease-specific (needing to have a certain severity of COPD), capability-related (needing to be cognitively capable, able to read and write, understand certain language, willing or able to provide consent), age-related (needing to have a minimum or maximum age), smoking history–related (being a current or former smoker), and technology-related (needing to have digital skills, internet access, own a certain device). More details about the concept of the self in self-management are provided in Multimedia Appendix 6.

#### Intended Population

**Figure 4:**
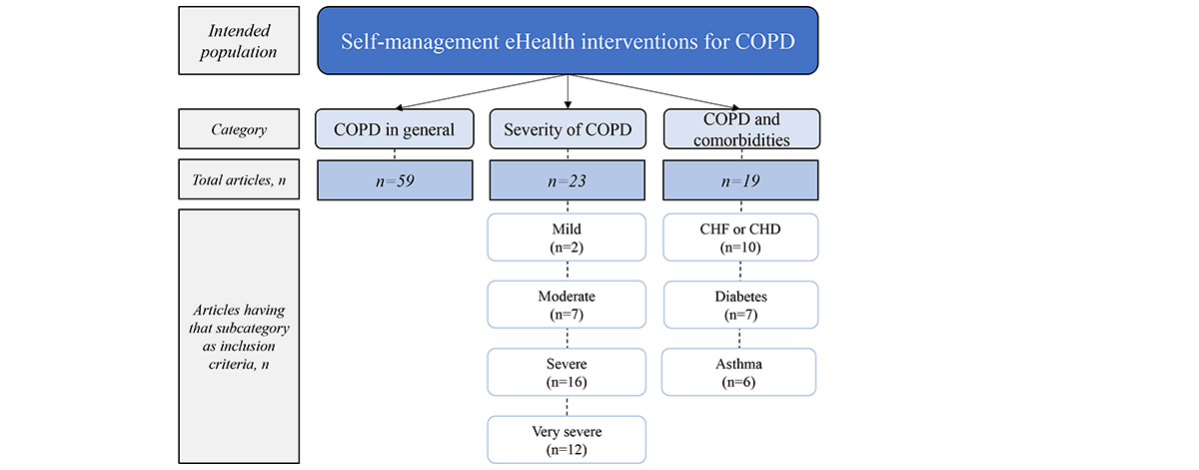
Intended population. CHD: chronic heart disease; CHF: chronic heart failure; COPD: chronic obstructive pulmonary disease.

There was some variation in the specific intended populations targeted in the articles. As shown in Figure 4, most studies (59/101, 58.4%) were targeted at persons with COPD in general, with 23 (22.8%) focusing on ≥1 specific COPD severities and 19 (18.8%) focusing on COPD in combination with other chronic conditions. Some articles included >1 comorbidity.

#### Included Population

**Figure 5:**
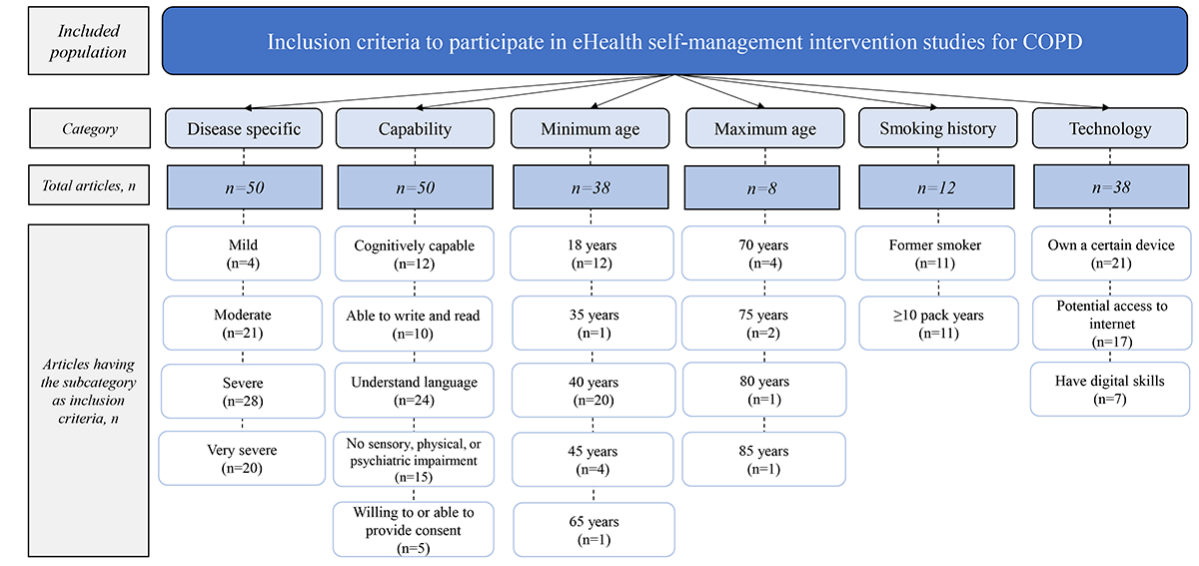
Included population. COPD: chronic obstructive pulmonary disease.

Figure 5 presents an overview of the identified included population. More studies (50/101, 49.5%) than outlined in the Intended Population section (23/101, 22.8%) had disease-specific inclusion criteria (focusing on ≥1 COPD severities). Of the 101 articles, 50 (49.5%) had capability-related inclusion criteria, requiring participants to, for example, be cognitively capable or able to write and read to be eligible for participation. Furthermore, in 37.6% (38/101) of the articles, participants needed to have a certain minimum age, with 40 years being the most common. In 7.9% (8/101) of the articles, the age needed to be below a certain maximum. The maximum age of 70 years was the most commonly mentioned inclusion criterion, cited 4 times. A total of 12 (11.9%) out of 101 articles had inclusion criteria regarding smoking (history) in which participants needed to be, for example, a former smoker. Finally, 38.6% (39/101) of the articles had technology-related inclusion criteria. Participants needed, for example, to own a smartphone or tablet and have digital skills to participate. Only 1 study explicitly mentioned having no exclusion criteria based on age, comorbidities, and previous participation in pulmonary rehabilitation. Furthermore, in the same study, participants did not need to have previous experience using digital technology.

#### Actual Population

**Figure 6:**
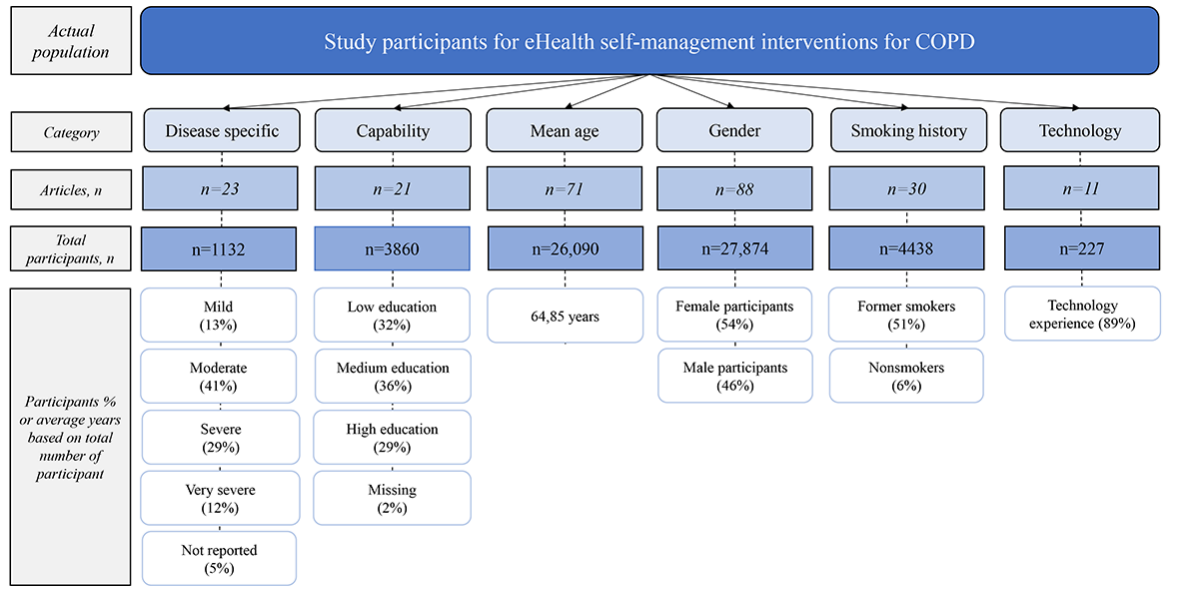
Actual population. COPD: chronic obstructive pulmonary disease.

Figure 6 shows the actual population included in the studies. In 24.7% (25/101) of the articles that mentioned the severity of their participants, most participants had moderate or severe COPD. Out of the 101 articles, only 21 (20.8%) shared a clear description of the education level of their participants, which were then categorized for this study. The educational level of participants could be categorized as low, medium, and high, which were almost equally distributed. Of the 70.3% (71/101) of the articles that shared the mean age of their participants, we calculated the combined mean age, which resulted in 64.85 years. The gender of participants was clearly mentioned in 88 (87.1%) out of 101 articles and was almost equally distributed. In 29.7% (30/101) of the articles that shared the smoking history of their participants, almost half of the participants (51%) were reported as current or former smokers. In 10.9% (11/101) of the articles that described technology-related experience, 89% of the participants had experience with technology.

### The Management in Self-Management

This section describes which self-management processes and BCTs were found within the different eHealth technologies. Details about this section are provided in Multimedia Appendix 7 (overview of self-management processes) and Multimedia Appendix 8 (overview of BCTs).

#### Self-Management Processes

Table 5 shows the self-management processes found in the articles. No article explicitly described which self-management processes were reflected in the intervention design. When analyzing how self-management processes were supported within the different studies, we identified that most studies (94/101, 93.1%) addressed the process of taking ownership toward health needs (eg, by including self-monitoring of symptoms or setting goals). Of the 101 included studies, 71 (70.3%) focused on the process of learning (eg, by including education within their technology), 27 (26.7%) on health care resources (eg, by enabling communication with health care professionals within the technology), 23 (22.8%) on performing health promotion activities (eg, by performing exercise or skill training), 17 (16.8%) on social resources (eg, by involving caregiver and family or peer-to-peer support), 1 (1%) on adjusting (eg, ways to cope), and 1 (1%) on integrating illness into daily life (eg, alternating daily lives to conserve energy). We found no eHealth technologies specifically focusing on the self-management processes: meaning making, spiritual resources, psychological resources, processing emotions, or community resources.

**Table 5:** Self-management processes within eHealth technologies. Each process is counted separately in this figure resulting in 234 self-management processes within 101 articles. “Meaning making,” “spiritual resources,” “psychological resources,” “processing emotions,” and “community resources” were not displayed, as these processes were not included.

Self-management process	Frequency, n (%)
Taking ownership of health needs	94 (40.2)
Learning	71 (30.3)
Health care resources	27 (11.5)
Performing health promotion activities	23 (9.8)
Social resources	17 (7.3)
Adjusting	1 (0.4)
Integrating illness into daily life	1 (0.4)

#### BCTs Used

Table 6 shows the BCTs extracted in this study. Only 2 (2%) out of 101 studies explicitly stated which BCTs were used. When analyzing the descriptions in the studies, we identified that feedback and monitoring were mostly used in the different articles (88/101, 87.1%; eg, monitoring activity status). This was followed by shaping knowledge (66/101, 65.3%; eg, receiving education), goals and planning (38/101, 37.6%; eg, action planning), associations (23/101, 22.8%; eg, receiving status updates), social support (14/101, 13.7%; eg, communication with other people with COPD), regulation (11/101, 10.9%; eg, addressing medication adherence), repetition and substitution (10/101, 9.9%; eg, habit formation), rewards and threat (6/101, 5.9%; eg, receiving visual rewards), natural consequences (5/101, 4.9%; eg, information about health consequences), self-belief (5/101, 4.9%; eg, increasing self-efficacy), comparison of behavior (5/101, 4.9%; eg, follow along exercise video), comparison of outcomes (2/101, 2%; eg, information about the effect of physical activity), antecedents (1/101, 1%; eg, adding objects to the environment), and identity (1/101, 1%; eg, prompt identification as a role model). The BCTs of covert learning and scheduled consequences were not observed in the studies.

**Table 6:** Behavior change techniques within eHealth technologies. Each technique was counted separately in this figure. Resulting in 275 behavior change techniques within 101 articles. “Scheduled consequences” and “covert learning” were not displayed, as these processes were not included.

Behavior change technique	Frequency, n (%)
Feedback and monitoring	88 (32)
Shaping knowledge	66 (24)
Goals and planning	38 (13.8)
Associations	23 (8.4)
Social support	14 (5.1)
Regulation	11 (4)
Repetition and substitution	10 (3.6)
Rewards and threat	6 (2.2)
Natural consequences	5 (1.8)
Comparison of behavior	5 (1.8)
Self-belief	5 (1.8)
Comparison of outcomes	2 (0.7)
Identity	1 (0.4)
Antecedents	1 (0.4)

## Discussion

### Principal Findings

This scoping review outlines the state of the art of eHealth self-management interventions for COPD. In the current literature, most eHealth technologies for COPD self-management focus on the physical aspect of self-management. eHealth technologies that include other aspects (eg, the social or mental aspects) are currently underrepresented in the literature. Moreover, it appeared that although eHealth interventions often aimed to target the whole COPD population, mostly only a subgroup of the COPD population was represented within the eHealth technology studies.

### Self-Management of COPD

#### Underlying Theories, Techniques, and Processes

Only a few studies (32/101, 31.7%) reported on using underlying theories and specific BCTs supporting their self-management eHealth interventions. No article explicitly mentioned focusing on certain self-management processes. This is surprising, given the fact that all studies aim to improve self-management and thus aim to achieve some sort of behavioral change. As the concept of self-management varies in the literature, reporting on the use of such processes, techniques, and theories may be beneficial for understanding underlying structures and processes that will initiate behavior change to improve self-management. Building on these processes, techniques, and theories and providing more detailed reports on what was perceived as useful, beneficial, and desirable for this target population can help advance the field and contribute to the existing body of work. This may simultaneously be valuable for informing future eHealth self-management initiatives as they can take into account these theories, techniques, and processes in their developments. When looking at the literature, the lack of reporting on underlying theories was prevalent in the review of Heimer et al [140], in which only 3 of the included studies reported specific BCTs. Furthermore, other studies encountered the problem of low reporting on BCTs; Hardeman et al [141] and Lorencatto et al [142] concluded that fewer than half of the planned BCTs were specified in the final published articles. In addition, a review from de Bruin et al [143] revealed that reporting about the active content of behavioral interventions varies considerably between studies. This limits the readers’ ability to compare, interpret, and generalize the effects of these studies [143]. Thus, including such theories in eHealth interventions and transparency in later reporting may lead to opportunities for achieving sustainable behavioral change.

#### The Physical Aspects of Self-Management

As we found in this review, there is a tendency toward managing the physical aspect of one’s disease in current eHealth technologies for COPD self-management. This is reflected throughout the different findings of this review. First, the functionality “self-monitoring” and the BCT “feedback and monitoring” were most often addressed. Although self-monitoring is very valuable and exacerbations may be detected at an early stage, this, nonetheless, demonstrates that the main focus lies on what happens with or inside the body. Second, the dominant physical aspect also manifests itself when looking at the self-management processes that are supported by the different technologies: “psychological resources,” “spiritual resources,” and “community resources” were not found to be included. The self-management process of “taking ownership of health needs” was mostly present, followed by “learning.” However, other processes, such as “integrating illness into daily life” and “adjusting,” were only observed once, although the target group had to deal with these aspects every single day [144]. We believe that not addressing these processes is a missed opportunity, given that supporting people with COPD during their day-to-day activities might lead to even better improved outcomes of self-management. Finally, the dimensions of bodily functioning and daily functioning were most frequently used. This illustrates the current underrepresentation of other dimensions within current eHealth technologies for COPD self-management. Other dimensions (eg, participation and mental well-being) were not as dominantly represented or not observed at all (eg, meaningfulness and quality of life). As we observed a small increase in dimensions over the past few years, we might notice a small change of focus. However, this is not as fundamental and still leaves a lot of room for improvement on this matter. When examining other chronic diseases (eg, rheumatoid arthritis), a review by Seppen et al [145] identified 4 different types of eHealth interventions used in the included articles. Although not explicitly stated, interventions were all related to the physical aspect (ie, medication adherence, activity plan, information, disease monitoring, and activity monitoring) [145]. Thus, the tendency of the physical domain may not only be limited to COPD. Therefore, future studies should investigate whether this view is also present in other chronic diseases.

### Inclusiveness and Representation of People With COPD

As all eHealth technologies target people with COPD as end users, only 14 (13.9%) out of 101 articles reported involving the patient perspective in their design or development process. This raised the question of whether and how the needs and perspectives of patients were taken into account. As including the perspective of end users leads to a better fit and increases the chances of successful adoption and sustained use [146], researchers should consider using such design principles when developing future eHealth technologies. This may provide many opportunities for improvements in self-management eHealth technologies for COPD.

Furthermore, it appeared that although articles outlined the target group to be the general population of people with COPD, they often recruited a specific subset of people with COPD. Certain inclusion criteria are made within the studies (eg, needing to own a smartphone and needing to have a certain disease severity). The consequence of such inclusion criteria leads to a restriction, in that only a selected group of individuals are included in the studies. While this may be due to practicalities (eg, the complexity of COPD as a progressive lung disease), the question remains whether the intervention is generalizable or applicable to the wider population of people with COPD, especially if they were not part of the studies in the first place. This is particularly relevant as there is no golden standard to determine, for example, when someone is considered too old to participate, and opinions on such matters are likely to vary widely among researchers. Even when the restricting the patient group may be justified for the study purposes, it still affects the generalizability of study results. Therefore, awareness and transparency should be provided regarding these potential restrictions related to the patient group within such studies.

Given that the most often used device is a (smart) measurement device (in combination with), a smartphone, or tablet, the group of people eligible to use these technologies in daily life is further limited. Effectively, this means that certain groups of people (eg, those who lack resources to buy these devices) are not included in the research and, therefore, the intervention might not be tested on people who are unfamiliar with smart devices or have low digital literacy. Previous studies showed that moderate levels of eHealth literacy and low levels of health literacy are prevalent among the COPD population [32,33]. Thus, we cannot assume that this population has access to eHealth technologies and has mastered the skills to manage such interventions without any support. Therefore, guidance should be made available to help those who need support in using these eHealth technologies. Furthermore, although there might be some practical reasoning behind the inclusion criteria (eg, lack of budget to provide devices for all participants), it may simultaneously widen the gap between people included in eHealth technology studies and the group of people who might need the support the most. This, in turn, could make health care accessible only to those with high (digital) literacy and those already equipped with the necessary resources to improve their health. This is most likely an unintentional and undesirable direction to head, but without proper awareness, it may easily become the blind spot in current and future eHealth intervention studies. Future studies must, therefore, be aware of possible subgroups, make efforts to include the underprivileged population, and be transparent in their research regarding the population reached. While we acknowledge the challenge of successfully recruiting participants who are representative of the population as a whole and recognize that this is extremely difficult to achieve, we nevertheless recommend that future studies strive to reach those people who are underrepresented and difficult to reach.

### Strengths and Limitations

This review provides a very first overview and a diverse insight into different underlying components of self-management eHealth interventions for COPD. It highlights the existing gaps in the literature and uncovers opportunities for the development of eHealth self-management interventions. To the best of our knowledge, no research has yet examined all these various aspects.

However, this review also has its limitations. First, data extraction and categorization were challenging due to the style of writing in articles (which comes with certain formats and word count limitations), the lack of explicit reporting on certain aspects, and the overlap between some processes and dimensions. It might be the case that through incomplete reporting in articles, certain self-management processes, BCTs, health dimensions, aspects of the technology, or details about the “self” could not be extracted. However, albeit being a scoping review, we followed a very systematic approach to mitigate this limitation as much as possible. Therefore, we believe that we were able to give a complete picture of the current literature about eHealth technologies for COPD self-management.

Second, some dimensions of positive health and self-management processes are closely related, intertwined with, or support each other. For example, the dimension of “quality of life” was not observed to be explicitly addressed within the eHealth interventions. However, studies may have an overarching goal of increasing the quality of life of people through the use of their intervention. One should be aware of this when interpreting these results, as the quality of life of people with COPD might still be affected by the use of eHealth intervention.

Third, most articles (470/588, 80%) were screened by 1 reviewer, leaving room for potential subjective judgment. However, by implementing several measures to ensure alignment (eg, extensive discussion of eligibility criteria, 20% of the screening conducted by 2 reviewers, and discussions to resolve discrepancies), we minimized possible implicit biases.

Finally, this scoping review investigated a broad range of aspects to grasp the state of the art, but not all. For example, this paper did not assess the effectiveness or impact of the interventions, and it also did not evaluate the quality or strength of the evidence. As this is a relatively new area of research, we should map the existing literature first. Furthermore, this review included only English articles, used COPD as a search term while it included a broad spectrum of lung diseases (such as emphysema and chronic bronchitis), and had a search date limitation. Consequently, some studies might have been missed, or some aspects may not have been investigated. However, to the best of our ability, we tried to provide a first overview while leaving opportunities for future research to focus on the aspects that were not covered within this scoping review. As such, this state-of-the-art overview could serve as a starting point for future systematic reviews and original research that will dive into more specific research areas.

### Conclusions

This scoping review provides an overview of the state-of-the-art eHealth technologies for COPD self-management interventions. We showed that current eHealth technologies tend to address the physical aspect of COPD self-management. These findings reveal a gap in the available literature, as many dimensions of the positive health paradigm and self-management processes are not addressed in current eHealth interventions for COPD self-management. However, as COPD is a chronic disease and exerts its impact on all aspects of one’s life, the underrepresented dimensions and processes might be very important to include. This might give people with COPD the tools needed to be able to adapt toward a new balance in life, and this would consider the person as a whole instead of only the bodily representation in the context of a disease. Our review also showcases another gap, namely, the effect of inclusion criteria that leads to a subgroup of people with COPD included in eHealth technology studies. Therefore, one should be cautious when interpreting results, as this may give a distorted view of the COPD population within these studies. These gaps demonstrate the need for more inclusive research and design of eHealth self-management interventions for people with COPD, focusing on multiple dimensions of the health paradigm. Future work should, therefore, go beyond the physical dimension and focus on including individuals in research who could benefit most from eHealth self-management interventions.

## Data Availability

All data generated or analyzed during this study are included in this published article and its supplementary information files.

## Appendix

Multimedia Appendix 1 PRISMA-ScR (Preferred Reporting Items For Systematic Reviews and Meta-Analyses extension for Scoping Reviews) checklist.

Multimedia Appendix 2 Search string.

Multimedia Appendix 3 Overview of the extraction and charting details.

Multimedia Appendix 4 Overview of the functionality and modality of eHealth interventions.

Multimedia Appendix 5 Overview of the positive health dimensions.

Multimedia Appendix 6 Overview of the self.

Multimedia Appendix 7 Overview of the self-management processes.

Multimedia Appendix 8 Overview of the behavior change techniques.

## Abbreviations

BCT: behavior change technique
COPD: chronic obstructive pulmonary disease
PRISMA-ScR: Preferred Reporting Items for Systematic Reviews and Meta-Analyses extension for Scoping Reviews
TRL: technology readiness level
