# Supplementary material for: The State of the Art of eHealth Self-Management Interventions for People With Chronic Obstructive Pulmonary Disease: Scoping Review
Source: J Med Internet Res. 2025 Mar 10;27:e57649. doi: 10.2196/57649 (PMC11933764; doi:10.2196/57649)
Supplement: Multimedia Appendix 2 [file jmir_v27i1e57649_app2.docx]

## Multimedia appendix 2. Search string.

**Article title:** The state-of-the-art of eHealth self-management interventions for people with Chronic Obstructive Pulmonary Disease: a scoping review

**Journal name:** Journal of Healthcare Informatics Research

**Author names:** Eline te Braake¹^,^ ², Roswita M. E. Vaseur², Christiane Grünloh¹^,^ ², Monique Tabak²

**Affiliation**s**:** ^1^Roessingh Research and Development, Enschede, the Netherlands; ^2^University of Twente, Biomedical Signals and Systems group, Faculty of Electrical Engineering, Mathematics, and Computer Science, Enschede, the Netherlands

**Correspondence:** Eline te Braake, Roessingh Research and Development, Roessinghsbleekweg 33b, 7522AH Enschede, the Netherlands, Tel +31 (0)88 087 5734. Email: [e.tebraake@rrd.nl](mailto:e.tebraake@rrd.nl)

| **Topic** | **Search** | **Query** |
| --- | --- | --- |
|  | #4 | #1 AND #2 AND #3 |
| Self-management | #3 | “Self-manag*” OR “Self manag*” OR "self-care" OR “Self care” OR "self-guidance" OR "self-control" OR "self-regulation" OR "self-inspection" OR "self-monitor*" OR “Self monitor*” OR "self-supervision" OR "self-government" OR "self-rule" |
| COPD | #2 | "Pulmonary Disease, Chronic Obstructive" OR "COPD" OR "chronic obstructive lung disease" OR "chronic obstructive pulmonary disease" OR "COAD" OR "chronic obstructive airway disease" OR "chronic obstructive pulmonary disease" OR "chronic airflow obstruction" |
| eHealth | #1 | "Telemedicine" OR "eHealth" OR "health technology" OR "mHealth" OR "m-health" OR "e-Health" OR "digital aid" OR "digital care" OR "Mobile Health" OR "Health, mobile" OR "e-mental health" OR "tele-health" OR "Telehealth" OR "Telecare" OR "Telemonitor*" OR "teleconsultation" OR "e-governance" OR "Mobile application*" |
