# Supplementary material for: The State of the Art of eHealth Self-Management Interventions for People With Chronic Obstructive Pulmonary Disease: Scoping Review
Source: J Med Internet Res. 2025 Mar 10;27:e57649. doi: 10.2196/57649 (PMC11933764; doi:10.2196/57649)
Supplement: Multimedia Appendix 3 [file jmir_v27i1e57649_app3.docx]

Multimedia appendix 3. Overview of extraction and charting details.

**Article title:** The state-of-the-art of eHealth self-management interventions for people with Chronic Obstructive Pulmonary Disease: a scoping review

**Journal name:** Journal of Healthcare Informatics Research

**Author names:** Eline te Braake¹^,^ ², Roswita M. E. Vaseur², Christiane Grünloh¹^,^ ², Monique Tabak²

**Affiliation**s**:** ^1^Roessingh Research and Development, Enschede, the Netherlands; ^2^University of Twente, Biomedical Signals and Systems group, Faculty of Electrical Engineering, Mathematics, and Computer Science, Enschede, the Netherlands

**Correspondence:** Eline te Braake, Roessingh Research and Development, Roessinghsbleekweg 33b, 7522AH Enschede, the Netherlands, Tel +31 (0)88 087 5734. Email: [e.tebraake@rrd.nl](mailto:e.tebraake@rrd.nl)

| Extraction categories | Extracted data | Reference | Way of extracting | Way of charting data |
| --- | --- | --- | --- | --- |
|  |  |  |  |  |
| **General** |  |  |  |  |
|  | a) Year of study  b) Type of study  c) Definition of self-management | n/a | Directly from data | Data clustered in bar graph [b], table [c], or presented descriptively [a] |
| **SQ1: What is the ‘e’ in eHealth self-management?** |  |  |  |  |
|  | a) Functionality  b) Modality  c) Technology Readiness (TRL) -level (The maturity of a technology)  d) eHealth development details | c) [42] | Both directly from data [a, b, d] and assessed/ categorized by the reviewer(s) [b, c] | Counted for separately [a, d] and available information about eHealth development status was categorized into the different TRL-levels [c]. Data is mapped into bar graph [b, c], table [d] or presented descriptively [a, d] |
| **SQ2: What is the ‘health’ in eHealth self-management?** |  |  |  |  |
|  | a) Positive health dimensions | a) [31] | Interpretation and assessed/ categorized by reviewer(s) | Each dimension is counted for separately and mapped in radar and bar chart |
| **SQ3: Who is the ‘self’ in self-management?** |  |  |  |  |
|  | a) Intended target population  b) Included target population  c) Actual target population | n/a | Both directly from data [a, b, c] and assessed/categorized by reviewer(s) [c] | When information on the education of participants is available in the demographics, this is categorized in low, medium, or high education [c] and clustered in a flow chart [a, b, c]. |
| **SQ4: What is the ‘management’ in eHealth self-management?** |  |  |  |  |
|  | a) Self-management processes  b) Behavioural change techniques (BCTs) | a) [20]  b) [21] | Both directly from data [b] and assessed/ categorized by the reviewer(s) [a, b] | Each process and behavioural change technique is counted for separately and mapped in a bar graph [a, b] |

## References

[20] D. Schulman-Green et al., Processes of Self-Management in Chronic Illness. J Nurs Scholarsh. 2012;44(2):136–144. doi: 10.1111/j.1547-5069.2012.01444.x.

[21] S. Michie et al., The behavior change technique taxonomy (v1) of 93 hierarchically clustered techniques: Building an international consensus for the reporting of behavior change interventions. Ann Behav Med. 2013;46(1):81–95. doi: 10.1007/s12160-013-9486-6.

[31] M. Huber et al., Towards a ‘patient-centred’ operationalisation of the new dynamic concept of health: a mixed methods study. BMJ Open. 2016;5:10091. doi: 10.1136/bmjopen-2015.

[42] S. Jansen-Kosterink, M. Broekhuis, and L. van Velsen. Time to act mature—Gearing eHealth evaluations towards technology readiness levels. Digit Health. 2022;8:29552976221113396. doi: 10.1177/20552076221113396.
