# Supplementary material for: The State of the Art of eHealth Self-Management Interventions for People With Chronic Obstructive Pulmonary Disease: Scoping Review
Source: J Med Internet Res. 2025 Mar 10;27:e57649. doi: 10.2196/57649 (PMC11933764; doi:10.2196/57649)
Supplement: Multimedia Appendix 8 [file jmir_v27i1e57649_app8.docx]

## Multimedia Appendix 8. Overview of Behavioral Change Techniques (BCTs)

**Article title:** The state-of-the-art of eHealth self-management interventions for people with Chronic Obstructive Pulmonary Disease: a scoping review

**Journal name:** Journal of Healthcare Informatics Research

**Author names:** Eline te Braake¹^,^ ², Roswita M. E. Vaseur², Christiane Grünloh¹^,^ ², Monique Tabak²

**Affiliation**s**:** ^1^Roessingh Research and Development, Enschede, the Netherlands; ^2^University of Twente, Biomedical Signals and Systems group, Faculty of Electrical Engineering, Mathematics, and Computer Science, Enschede, the Netherlands

**Correspondence:** Eline te Braake, Roessingh Research and Development, Roessinghsbleekweg 33b, 7522AH Enschede, the Netherlands, Tel +31 (0)88 087 5734. Email: [e.tebraake@rrd.nl](mailto:e.tebraake@rrd.nl)

| *Author* | *Reference* | *Antecedents* | *Associations* | *Comparison of behaviour* | *Comparison of outcomes* | *Covert learning* | *Feedback and monitoring* | *Goals and planning* | *Identity* | *Natural consequences* | *Regulation* | *Repetition and substitution* | *Rewards and threat* | *Scheduled consequences* | *Self-belief* | *Shaping knowledge* | *Social support* |
| --- | --- | --- | --- | --- | --- | --- | --- | --- | --- | --- | --- | --- | --- | --- | --- | --- | --- |
| Alcazar et al. (2016) | [76] | - | - | - | - | - | 🗸 | - | - | - | - | - | - | - | - | - | - |
| Alharbey et al. (2019) | [63] | - | - | - | - | - | 🗸 | - | - | - | - | - | 🗸 | - | - | 🗸 | - |
| Ali et al (2021) | [44] | - | - | - | - | - | 🗸 | - | - | - | - | - | - | - | - | 🗸 | 🗸 |
| Au et al. (2016) | [77] | - | - | - | - | - | 🗸 | - | - | - | - | - | - | - | - | 🗸 | - |
| Barenfeld et al. (2020) | [74] | - | - | - | - | - | 🗸 | 🗸 | - | - | - | - | - | - | - | 🗸 | 🗸 |
| Barenfeld et al. (2022) | [78] | - | - | - | - | - | 🗸 | 🗸 | - | - | - | - | - | - | - | 🗸 | 🗸 |
| Barken et al. (2018) | [79] | - | - | - | - | - | 🗸 | - | - | - | - | - | - | - | - | - | - |
| Bentley et al. (2020) | [72] | - | - | - | - | - | 🗸 | 🗸 | - | - | - | - | 🗸 | - | - | 🗸 | - |
| Benzo et al. (2018) | [80] | - | - | - | - | - | 🗸 | 🗸 | - | - | - | 🗸 | - | - | - | 🗸 | - |
| Billington et al. (2015) | [81] | - | - | - | - | - | - | 🗸 | - | - | 🗸 | - | - | - | 🗸 | 🗸 | - |
| Bødker et al. (2015) | [82] | - | - | - | - | - | - | - | - | - | - | 🗸 | - | - | - | - | - |
| Boer et al. (2018) | [83] | - | - | - | - | - | 🗸 | - | - | - | 🗸 | - | - | - | - | 🗸 | - |
| Boer et al. (2019) | [84] | - | - | - | - | - | 🗸 | - | - | - | 🗸 | - | - | - | - | 🗸 | - |
| Bourbeau et al. (2016) | [61] | - | - | - | - | - | 🗸 | - | - | - | - | - | - | - | - | 🗸 | - |
| Bugajski et al. (2019) | [68] | - | - | - | - | - | - | 🗸 | - | - | - | - | - | - | - | 🗸 | 🗸 |
| Burkow et al. (2013) | [85] | - | - | - | - | - | 🗸 | - | - | - | - | - | - | - | - | 🗸 | - |
| Burkow et al. (2015) | [86] | - | - | - | - | - | 🗸 | - | - | - | - | - | - | - | - | 🗸 | - |
| Burkow et al. (2018) | [75] | 🗸 | 🗸 | 🗸 | 🗸 | - | 🗸 | 🗸 | - | 🗸 | - | - | 🗸 | - | - | 🗸 | - |
| Cameron-Tucker et al. (2016) | [50] | - | - | - | - | - | - | 🗸 | - | - | - | - | - | - | - | - | - |
| Choi et al. (2021) | [45] | - | - | - | - | - | 🗸 | - | - | - | - | - | - | - | - | 🗸 | - |
| Cooper et al. (2022) | [87] | - | - | - | - | - | 🗸 | - | - | - | - | - | - | - | - | v | - |
| Coultas et al. (2018) | [64] | - | - | - | - | - | 🗸 | 🗸 | - | - | - | - | - | - | - | 🗸 | - |
| Coventry et al. (2019) | [65] | - | - | - | - | - | - | 🗸 | - | - | 🗸 | - | - | - | - | - | - |
| Criner et al. (2021) | [88] | - | - | - | - | - | 🗸 | - | - | - | - | - | - | - | - | - | - |
| De San Miguel et al. (2013) | [89] | - | - | - | - | - | 🗸 | - | - | - | - | - | - | - | - | - | - |
| Deng et al. (2020) | [90] | - | - | - | - | - | 🗸 | - | - | - | - | - | - | - | - | 🗸 | - |
| Dhadge et al. (2020) | [91] | - | - | - | - | - | 🗸 | - | - | - | - | - | - | - | - | - | - |
| Doyle et al. (2021) | [49] | - | 🗸 | - | - | - | 🗸 | 🗸 | - | - | - | - | - | - | - | 🗸 | 🗸 |
| Early et al. (2017) | [92] | - | - | - | - | - | 🗸 | 🗸 | - | - | - | - | - | - | - | 🗸 | - |
| Farias et al. (2019) | [93] | - | 🗸 | - | - | - | 🗸 | - | - | - | - | - | - | - | - | - | - |
| Farmer et al. (2017) | [51] | - | - | - | - | - | 🗸 | - | - | - | - | - | - | - | - | 🗸 | - |
| Fitzsimmons et al. (2016) | [94] | - | - | - | - | - | 🗸 | - | - | - | - | - | - | - | - | - | - |
| Haesum et al. (2012) | [95] | - | - | - | - | - | 🗸 | - | - | - | - | - | - | - | - | - | - |
| Hardinge et al. (2015) | [52] | - | - | - | - | - | 🗸 | 🗸 | - | - | - | - | - | - | - | 🗸 | - |
| Hoaas et al. (2016) | [69] | - | - | - | - | - | 🗸 | - | - | - | - | - | - | - | - | - | - |
| Hoaas et al. (2016) | [96] | - | - | - | - | - | 🗸 | - | - | - | - | - | - | - | - | - | - |
| Houchen-Wolloff et al. (2021) | [97] | - | - | - | - | - | 🗸 | 🗸 | - | - | - | - | - | - | - | 🗸 | 🗸 |
| Huniche et al. (2013) | [98] | - | - | - | - | - | 🗸 | - | - | - | - | - | - | - | - | - | - |
| Jolly et al. (2018) | [66] | - | - | - | - | - | 🗸 | - | - | - | - | - | - | - | - | 🗸 | - |
| Kargiannakis et al. (2017) | [99] | - | - | - | - | - | 🗸 | - | - | - | - | - | - | - | - | - | - |
| Kaye et al. (2021) | [100] | - | 🗸 | - | - | - | 🗸 | - | - | - | - | 🗸 | - | - | - | 🗸 | - |
| Kessler et al. (2018) | [62] | - | - | - | - | - | 🗸 | 🗸 | - | - | - | - | - | - | - | 🗸 | - |
| Kjellsdotter et al. (2021) | [101] | - | - | - | - | - | - | - | - | - | - | - | - | - | - | 🗸 | - |
| Knox et al. (2021) | [102] | - | - | - | - | - | 🗸 | - | - | - | - | - | - | - | - | - | - |
| Koff et al. (2021) | [103] | - | - | - | - | - | 🗸 | - | - | - | - | - | - | - | - | 🗸 | - |
| Kooij et al. (2021) | [104] | - | - | - | - | - | 🗸 | 🗸 | - | - | - | - | - | - | - | 🗸 | - |
| Korpershoek et al. (2020) | [47] | - | 🗸 | - | 🗸 | - | 🗸 | 🗸 | - | 🗸 | - | 🗸 | 🗸 | - | - | - | 🗸 |
| Korpershoek et al. (2020) | [46] | - | - | - | - | - | 🗸 | - | - | - | - | - | - | - | - | 🗸 | - |
| Lee et al. (2012) | [105] | - | - | - | - | - | 🗸 | - | - | - | - | - | - | - | - | 🗸 | - |
| Lilholt et al. (2015) | [106] | - | - | - | - | - | 🗸 | - | - | - | - | - | - | - | - | 🗸 | - |
| Lundell et al. (2020) | [25] | - | - | - | - | - | 🗸 | - | - | - | - | - | - | - | - | - | - |
| Maathuis et al. (2014) | [107] | - | - | - | - | - | 🗸 | 🗸 | - | - | - | - | - | - | - | - | - |
| Mark et al. (2013) | [108] | - | - | - | - | - | - | - | - | - | - | 🗸 | - | - | - | 🗸 | - |
| Marklund et al. (2021) | [26] | - | 🗸 | - | - | - | 🗸 | - | - | - | - | - | - | - | - | 🗸 | - |
| Marquis et al. (2015) | [109] | - | - | - | - | - | - | - | - | - | - | 🗸 | - | - | - | 🗸 | - |
| Mathar et al. (2015) | [110] | - | - | - | - | - | 🗸 | - | - | - | 🗸 | - | - | - | - | - | - |
| Mierdel et al. (2015) | [111] | - | - | - | - | - | 🗸 | 🗸 | - | - | - | - | - | - | - | 🗸 | - |
| Miller et al. (2021) | [70] | - | 🗸 | - | - | - | 🗸 | - | - | - | - | - | - | - | - | - | - |
| Nield et al. (2012) | [112] | - | - | - | - | - | 🗸 | - | - | - | - | 🗸 | - | - | - | 🗸 | - |
| North et al. (2020) | [113] | - | 🗸 | - | - | - | - | - | - | - | - | - | - | - | - | 🗸 | - |
| Nyberg et al. (2019) | [114] | - | - | - | - | - | 🗸 | - | - | - | - | - | - | - | - | 🗸 | - |
| Orme et al. (2018) | [115] | - | 🗸 | - | - | - | 🗸 | - | - | - | - | - | - | - | - | 🗸 | - |
| Park et al. (2020) | [67] | - | - | - | - | - | 🗸 | 🗸 | - | - | - | 🗸 | 🗸 | - | - | 🗸 | 🗸 |
| Patel et al. (2021) | [116] | - | 🗸 | - | - | - | 🗸 | - | - | - | 🗸 | - | - | - | - | - | - |
| Rassouli et al. (2018) | [117] | - | - | - | - | - | 🗸 | - | - | - | - | 🗸 | - | - | - | 🗸 | - |
| Rixon et al. (2017) | [118] | - | - | - | - | - | 🗸 | - | - | - | - | - | - | - | - | 🗸 | - |
| Robinson et al. (2020) | [73] | - | - | - | - | - | 🗸 | 🗸 | - | - | - | - | - | - | - | 🗸 | 🗸 |
| Rodriguez Hermosa et al. (2020) | [119] | - | 🗸 | - | - | - | 🗸 | - | - | - | - | - | - | - | - | 🗸 | - |
| Schnoor et al. (2022) | [120] | - | - | 🗸 | - | - | 🗸 | - | - | - | 🗸 | - | - | - | - | 🗸 | - |
| Sheridan et al. (2020) | [121] | - | - | - | - | - | 🗸 | 🗸 | - | - | - | - | - | - | - | 🗸 | - |
| Sieverink et al. (2019) | [122] | - | - | - | - | - | 🗸 | 🗸 | - | - | - | - | - | - | - | 🗸 | - |
| Sloots et al. (2021) | [27] | - | 🗸 | - | - | - | 🗸 | - | - | - | 🗸 | - | - | - | - | - | - |
| Stamenova et al. (2020) | [123] | - | 🗸 | - | - | - | 🗸 | 🗸 | - | - | - | - | - | - | - | 🗸 | - |
| Steventon et al. (2013) | [124] | - | - | - | - | - | - | 🗸 | - | - | 🗸 | - | - | - | - | 🗸 | 🗸 |
| Talboom-Kamp et al. (2017) | [125] | - | 🗸 | - | - | - | 🗸 | 🗸 | - | - | - | - | - | - | - | 🗸 | - |
| Talboom-Kamp et al. (2017) | [126] | - |  | - | - | - | 🗸 | 🗸 | - | - | - | - | - | - | - | 🗸 | - |
| Talboom-Kamp et al. (2019) | [127] | - | 🗸 | - | - | - | 🗸 | - | - | - | - | - | - | - | - | 🗸 | - |
| Tabak et al. (2014) | [128] | - | 🗸 | - | - | - | 🗸 | 🗸 | - | - | - | - | - | - | - | 🗸 | - |
| ter Stal et al. (2021) | [129] | - | 🗸 | 🗸 | - | - | 🗸 | 🗸 | - | - | - | - | - | - | - | 🗸 | - |
| Thomas et al. (2017) | [71] | - | - | 🗸 | - | - | 🗸 | - | - | - | - | 🗸 | - | - | - | 🗸 | - |
| van Buul et al. (2018) | [130] | - | - | - | - | - | 🗸 | 🗸 | - | - | - | - | - | - | - | 🗸 | - |
| van Buul et al. (2021) | [131] | - | 🗸 | - | - | - | 🗸 | - | - | - | - | - | - | - | - | - | 🗸 |
| van der Heijden et al. (2013) | [132] | - | 🗸 | - | - | - | 🗸 | - | - | - | - | - | - | - | - | - | - |
| van der Weegen et al. (2013) | [43] | - | - | - | - | - | 🗸 | 🗸 | - | - | - | - | 🗸 | - | - | - | - |
| van der Weegen et al. (2015) | [55] | - | - | - | - | - | 🗸 | 🗸 | - | - | - | - | - | - | - | - | - |
| van Lieshout et al. (2020) | [133] | - | 🗸 | - | - | - | 🗸 | - | - | - | - | - | - | - | - | - | - |
| van Zelst et al. (2021) | [134] | - | - | - | - | - | 🗸 | - | - | - | - | - | - | - | - | 🗸 | - |
| Vatnøy et al. (2017) | [135] | - | - | - | - | - | 🗸 | - | - | - | - | - | - | - | - | - | - |
| Velardo et al. (2017) | [53] | - | - | - | - | - | 🗸 | - | - | - | - | - | - | - | - | - | - |
| Verwey et al. (2014) | [56] | - | - | - | - | - | 🗸 | 🗸 | - | - | - | - | - | - | - | - | - |
| Voncken-Brewster et al. (2013) | [57] | - | - | - | - | - | 🗸 | 🗸 | 🗸 | 🗸 | - | - | - | - | - | - | 🗸 |
| Voncken-Brewster et al. (2014) | [59] | - | - | - | - | - | 🗸 | 🗸 | - | - | 🗸 | - | - | - | 🗸 | 🗸 | - |
| Voncken-Brewster et al. (2015) | [58] | - | 🗸 | - | - | - | 🗸 | 🗸 | - | 🗸 | - | - | - | - | 🗸 | 🗸 | - |
| Voncken-Brewster et al. (2017) | [60] | - | - | - | - | - | 🗸 | 🗸 | - | 🗸 | 🗸 | - | - | - | 🗸 | 🗸 | 🗸 |
| Vorrink et al. (2017) | [136] | - | 🗸 | - | - | - | 🗸 | - | - | - | - | - | - | - | - | - | - |
| Walters et al. (2013) | [137] | - | - | - | - | - | - | 🗸 | - | - | - | - | - | - | - | - | - |
| Wang et al., (2021) | [48] | - | - | 🗸 | - | - | - | - | - | - | - | - | - | - | - | 🗸 | 🗸 |
| Whelan et al. (2019) | [54] | - | 🗸 | - | - | - | 🗸 | - | - | - | - | - | - | - | - | 🗸 | - |
| Williams et al. (2014) | [32] | - | - | - | - | - | 🗸 | - | - | - | - | - | - | - | - | 🗸 | - |
| Zanaboni et al. (2013) | [138] | - | - | - | - | - | 🗸 | - | - | - | - | - | - | - | - | - | - |
| Zanaboni et al. (2017) | [139] | - | - | - | - | - | 🗸 | - | - | - | - | - | - | - | 🗸 | 🗸 | - |
| *Total* |  | *1* | *23* | *5* | *2* | *0* | *88* | *38* | *1* | *5* | *11* | *10* | *6* | *0* | *5* | *66* | *14* |

## References

[25] S. Lundell, M. Modig, Å. Holmner, and K. Wadell. Perceptions of home telemonitoring use among patients with chronic obstructive pulmonary disease: Qualitative study. JMIR Mhealth Uhealth. 2020;8(6):e16343. doi: 10.2196/16343.

[26] Marklund S, Tistad M, Lundell S, Östrand L, Sörlin A, Boström C, et al. Experiences and factors affecting usage of an eHealth tool for self-management among people with chronic obstructive pulmonary disease: qualitative study. J Med Internet Res. Apr 30, 2021;23(4):e25672

[27] J. Sloots et al., Adherence to an ehealth self-management intervention for patients with both copd and heart failure: Results of a pilot study. Int J Chron Obstruct Pulmon Dis. 2021;16:2089–2103. doi: 10.2147/COPD.S299598.

[32] V. Williams, J. Price, M. Hardinge, L. Tarassenko, and A. Farmer. Using a mobile health application to support self-management in COPD: A qualitative study. Br J Gen Practi. 2014 Jul;64(624):e392-400. doi: 10.3399/bjgp14X680473

[43] S. Van Der Weegen, R. Verwey, M. Spreeuwenberg, H. Tange, T. Van Der Weijden, and L. De Witte. The development of a mobile monitoring and feedback tool to stimulate physical activity of people with a chronic disease in primary care: A user-centered design. JMIR Mhealth Uhealth. 2013;1(2). doi: 10.2196/mhealth.2526.

[44] L. Ali et al., Effects of Person-Centered Care Using a Digital Platform and Structured Telephone Support for People with Chronic Obstructive Pulmonary Disease and Chronic Heart Failure: Randomized Controlled Trial. J Med Internet Res. 2021;23(12). doi: 10.2196/26794.

[45] J. Y. Choi, M. George, and S. Y. Yun. Development of a smartphone application for Korean patients with chronic obstructive pulmonary disease: Self-monitoring based action plans. Appl Nurs Res. 2021;61:151475. doi: 10.1016/j.apnr.2021.151475.

[46] Y. J. G. Korpershoek, S. Hermsen, L. Schoonhoven, M. J. Schuurmans, and J. C. A. Trappenburg. User-centered design of a mobile health intervention to enhance exacerbation-related self-management in patients with chronic obstructive pulmonary disease (Copilot): Mixed methods study. J Med Internet Res. 2020;22(6). doi: 10.2196/15449.

[47] Y. J. G. Korpershoek, T. Holtrop, S. C. J. M. Vervoort, L. Schoonhoven, M. J. Schuurmans, and J. C. A. Trappenburg. Early-stage feasibility of a mobile health intervention (copilot) to enhance exacerbation-related self-management in patients with chronic obstructive pulmonary disease: Multimethods approach. JMIR Form Res. 2020;4(11). doi: 10.2196/21577.

[48] L. H. Wang, Y. M. Guo, M. Wang, and Y. Zhao. A mobile health application to support self-management in patients with chronic obstructive pulmonary disease: a randomised controlled trial. Clin Rehabil. 2021;35(1);90–101. doi: 10.1177/0269215520946931.

[49] J. Doyle et al., A Digital Platform to support Self-management of Multiple Chronic Conditions (ProACT): Findings in Relation to Engagement During a One-Year Proof-of-Concept Trial. J Med Internet Res. 2021;23(12). doi: 10.2196/22672.

[50] H. L. Cameron-Tucker, R. Wood-Baker, L. Joseph, J. A. Walters, N. Schüz, and E. H. Walters. A randomized controlled trial of telephone-mentoring with home-based walking preceding rehabilitation in COPD. Inter J Chron Obstruct Pulmon Dis. 2016;11(1):1991–2000. doi: 10.2147/COPD.S109820.

[51] A. Farmer et al., Self-Management Support Using a Digital Health System Compared With Usual Care for Chronic Obstructive Pulmonary Disease: Randomized Controlled Trial. J Med Internet Res. 2017;19(5). doi: 10.2196/jmir.7116.

[52] M. Hardinge et al., Using a mobile health application to support self-management in chronic obstructive pulmonary disease: A six-month cohort study . BMC Med Inform Decis Mak. 2015;15(1). doi: 10.1186/s12911-015-0171-5.

[53] C. Velardo et al., Digital health system for personalised COPD long-term management. BMC Med Inform Decis Mak. 2017;17(1). doi: 10.1186/s12911-017-0414-8.

[54] M. E. Whelan, C. Velardo, H. Rutter, L. Tarassenko, and A. J. Farmer. Mood monitoring over one year for people with chronic obstructive pulmonary disease using a mobile health system: Retrospective analysis of a randomized controlled trial. JMIR Mhealth Uhealth. 2019;7(11). doi: 10.2196/14946.

[55] S. Van Der Weegen, R. Verwey, M. Spreeuwenberg, H. Tange, T. Van Der Weijden, and L. De Witte. It’s LiFe! Mobile and web-based monitoring and feedback tool embedded in primary care increases physical activity: A cluster randomized controlled trial. J Med Internet Res. 2015;17(7). doi: 10.2196/jmir.4579.

[56] R. Verwey, S. van der Weegen, M. Spreeuwenberg, H. Tange, T. van der Weijden, and L. de Witte. A pilot study of a tool to stimulate physical activity in patients with COPD or type 2 diabetes in primary care. J Telemed Telecare. 2014;20(1):29–34, 2014. doi: 10.1177/1357633X13519057.

[57] V. Voncken-Brewster, A. Moser, T. van der Weijden, Z. Nagykaldi, H. de Vries, and H. Tange. Usability evaluation of an online, tailored self-management intervention for chronic obstructive pulmonary disease patients incorporating behavior change techniques. JMIR Res Protoc. 2013;2(1). doi: 10.2196/resprot.2246.

[58] V. Voncken-Brewster, H. Tange, H. de Vries, Z. Nagykaldi, B. Winkens, and T. van der Weijden. A randomized controlled trial evaluating the effectiveness of a web-based, computer-tailored self-management intervention for people with or at risk for COPD. Inter J Chron Obstruct Pulmon Dis. 2015;10:1061–1073. doi: 10.2147/COPD.S81295.

[59] V. Voncken-Brewster, H. Tange, A. Moser, Z. Nagykaldi, H. De Vries, and T. Van Der Weijden. Integrating a tailored e-health self-management application for chronic obstructive pulmonary disease patients into primary care: a pilot study. 2014. BMC Fam Pract. 2014;15:4. doi: 10.1186/1471-2296-15-4.

[60] V. Voncken-Brewster et al., The Impact of Participant Characteristics on Use and Satisfaction of a Web-Based Computer-Tailored Chronic Obstructive Pulmonary Disease Self-Management Intervention: A Process Evaluation. JMIR Form Res. 2017;1(1):e1. doi:10.2196//formative.6585.

[61] J. Bourbeau, P. Casan, S. Tognella, P. Haidl, J. B. Texereau, and R. Kessler. An international randomized study of a home-based self-management program for severe COPD: The COMET. Inter J Chron Obstruct Pulmon Dis. 2016;11(1):1447–1451. doi: 10.2147/COPD.S107151.

[62] R. Kessler et al., CoMET: A multicomponent home-based disease-management programme versus routine care in severe COPD. Eur Respir J. 2018;51(1). doi: 10.1183/13993003.01612-2017.

[63] R. Alharbey and S. Chatterjee. An mHealth assistive system ‘mylung’ to empower patients with chronic obstructive pulmonary disease: Design science research. JMIR Form Res. 2019;3(1). doi: 10.2196/12489.

[64] D. B. Coultas et al., Home-based physical activity coaching, physical activity, and health care utilization in chronic obstructive pulmonary disease chronic obstructive pulmonary disease self-management activation research trial secondary outcomes. Ann Am Thorac Soc. 2018;15(4):470–478. doi: 10.1513/AnnalsATS.201704-308OC.

[65] P. A. Coventry, A. Blakemore, E. Baker, M. Sidhu, D. Fitzmaurice, and K. Jolly, The Push and Pull of Self-Managing Mild COPD: An Evaluation of Participant Experiences of a Nurse-Led Telephone Health Coaching Intervention. Qual Health Res. 2019;29(5):658–671. doi: 10.1177/1049732318809679.

[66] K. Jolly et al., Self management of patients with mild COPD in primary care: Randomised controlled trial. BMJ (Online). 2018;361. doi: 10.1136/bmj.k2241.

[67] S. K. Park, C. H. Bang, and S. H. Lee. Evaluating the effect of a smartphone app-based self-management program for people with COPD: A randomized controlled trial. Appl Nurs Res. 2020;52. doi: 10.1016/j.apnr.2020.151231.

[68] A. Bugajski et al., Effects of a Digital Self-care Intervention in Adults with COPD: A Pilot Study. West J Nurs Res. 2020;42(9):736–746. doi: 10.1177/0193945919892282.

[69] H. Hoaas, H. K. Andreassen, L. A. Lien, A. Hjalmarsen, and P. Zanaboni. Adherence and factors affecting satisfaction in long-term telerehabilitation for patients with chronic obstructive pulmonary disease: A mixed methods study eHealth/ telehealth/ mobile health systems. BMC Med Inform Decis Mak. 2016;16(1). doi: 10.1186/s12911-016-0264-9.

[70] S. Miller et al., Feasibility of resp-fit: Technology-enhanced self-management intervention for adults with copd. Inter J Chron Obstruct Pulmon Di. 2021;16:3263–3273. doi: 10.2147/COPD.S326675.

[71] R. M. Thomas et al., Inhaler training delivered by internet-based home videoconferencing improves technique and quality of life. Respir Care. 2017;62(11):1412–1422. doi: 10.4187/respcare.05445.

[72] C. L. Bentley et al., The use of a smartphone app and an activity tracker to promote physical activity in the management of chronic obstructive pulmonary disease: Randomized controlled feasibility study. JMIR Mhealth Uhealth. 2020;8(6). doi: 10.2196/16203.

[73] S. A. Robinson, E. S. Wan, S. L. Shimada, C. R. Richardson, and M. L. Moy. Age and attitudes towards an internet-mediated, pedometer-based physical activity intervention for chronic obstructive pulmonary disease: Secondary analysis. JMIR Aging. 2020;3(2). doi: 10.2196/19527.

[74] E. Barenfeld, L. Ali, S. Wallström, A. Fors, and I. Ekman. Becoming more of an insider: A grounded theory study on patients’ experience of a person-centred e-health intervention. PLoS One. 2020;15(11). doi: 10.1371/journal.pone.0241801.

[75] T. M. Burkow, L. K. Vognild, E. Johnsen, A. Bratvold, and M. J. Risberg. Promoting exercise training and physical activity in daily life: A feasibility study of a virtual group intervention for behaviour change in COPD. BMC Med Inform Decis Mak. 2018;18(1). doi: 10.1186/s12911-018-0721-8.

[76] B. Alcazar et al., The evaluation of a remote support program on quality of life and evolution of disease in COPD patients with frequent exacerbations. BMC Pulm Med. 2016;16(1). doi: 10.1186/s12890-016-0304-3.

[77] D. H. Au, D. S. Macaulay, J. L. Jarvis, U. S. Desai, and H. G. Birnbaum. Impact of a telehealth and care management Program for patients with chronic obstructive pulmonary disease. Ann Am Thorac Soc. 2015;12(3):323–331. doi: 10.1513/AnnalsATS.201501-042OC.

[78] E. Barenfeld, J. M. Fuller, S. Wallström, A. Fors, L. Ali, and I. Ekman. Meaningful use of a digital platform and structured telephone support to facilitate remote person-centred care – a mixed-method study on patient perspectives. BMC Health Serv Res. 2022;22(1). doi: 10.1186/s12913-022-07831-8.

[79] T. L. Barken, E. Thygesen, and U. Söderhamn. Unlocking the limitations: Living with chronic obstructive pulmonary disease and receiving care through telemedicine—A phenomenological study. J Clin Nurs. 2018;27(1):132–142. doi: 10.1111/jocn.13857

[80] R. P. Benzo, K. M. Kramer, J. P. Hoult, P. M. Anderson, I. M. Begue, and S. J. Seifert. Development and feasibility of a home pulmonary rehabilitation program with health coaching. Respir Care. 2018;63(2):131–140. doi: 10.4187/respcare.05690.

[81] J. Billington, S. Coster, T. Murrells, and I. Norman. Evaluation of a nurse-led educational telephone intervention to support self-management of patients with chronic obstructive pulmonary disease: A randomized feasibility study. COPD: J of COPD. 2015;12(4):395–403. doi: 10.3109/15412555.2014.974735.

[82] M. Bødker and A. Juul Nielsen. Providing rehabilitation online – invisible work and diagnostic agents. J Health Organ Manag. 2015;29(7):948–964. doi: 10.1108/JHOM-06-2014-0091.

[83] L. M. Boer et al., Validation of ACCESS: An automated tool to support self-management of COPD exacerbations. Inter J of COPD. 2018;13:3255–3267. doi: 10.2147/COPD.S167272.

[84] L. Boer et al., A smart mobile health tool versus a paper action plan to support self-management of chronic obstructive pulmonary disease exacerbations: randomized controlled trial. JMIR Mhealth Uhealth. 2019;7(10):e14408, 2019, doi: 10.2196/14408

[85] T. M. Burkow et al., Internet-enabled pulmonary rehabilitation and diabetes education in group settings at home: A preliminary study of patient acceptability. BMC Med Inform Decis Mak. 2013;13(1). doi: 10.1186/1472-6947-13-33.

[86] T. M. Burkow et al., Comprehensive pulmonary rehabilitation in home-based online groups: A mixed method pilot study in COPD. BMC Res Notes. 2015;8(1). doi: 10.1186/s13104-015-1713-8.

[87] R. Cooper et al., Evaluation of myCOPD Digital Self-management Technology in a Remote and Rural Population: Real-world Feasibility Study. JMIR Mhealth Uhealth. 2022;10(2). doi: 10.2196/30782.

[88] G. J. Criner, T. Cole, K. A. Hahn, K. Kastango, J. M. Eudicone, and I. Gilbert. Use of a Digital Chronic Obstructive Pulmonary Disease Respiratory Tracker in a Primary Care Setting: A Feasibility Study. Pulm Ther. 2021;7(2):533–547. doi: 10.1007/s41030-021-00168-3.

[89] K. De San Miguel, J. Smith, and G. Lewin. Telehealth remote monitoring for community-dwelling older adults with chronic obstructive pulmonary disease. Telemed J E Health. 2013;19(9):652–657. doi: 10.1089/tmj.2012.0244.

[90] N. Deng et al., Using mobile health technology to deliver a community-based closed-loop management system for chronic obstructive pulmonary disease patients in remote areas of china: Development and prospective observational study. JMIR Mhealth Uhealth. 2020;8(11). doi: 10.2196/15978.

[91] N. Dhadge et al., Monitoring of inhaler use at home with a smartphone video application in a pilot study. NPJ Prim Care Respir Med. 2020;30(1). doi: 10.1038/s41533-020-00203-x.

[92] F. Early, J. S. Young, E. Robinshaw, E. Z. Mi, E. Z. Mi, and J. P. Fuld. A case series of an off-the-shelf online health resource with integrated nurse coaching to support self-management in COPD. Inter J Chron Obstruct Pulmon. Dis. 20217;12:2955–2967. doi: 10.2147/COPD.S139532.

[93] R. Farias et al., Innovating the treatment of COPD exacerbations: A phone interactive telesystem to increase COPD Action Plan adherence. BMJ Open Respir Res. 2019;6(1). doi: 10.1136/bmjresp-2018-000379.

[94] D. A. Fitzsimmons, J. Thompson, C. L. Bentley, and G. A. Mountain. Comparison of patient perceptions of Telehealth-supported and specialist nursing interventions for early stage COPD: A qualitative study. BMC Health Serv Res. 2016;16(1). doi: 10.1186/s12913-016-1623-z.

[95] L. K. E. Haesum et al., Cost-utility analysis of a telerehabilitation program: A case study of COPD patients. Telem E Health. 2012;18(9):688–692. doi: 10.1089/tmj.2011.0250.

[96] H. Hoaas, B. Morseth, A. E. Holland, and P. Zanaboni. Are Physical Activity and Benefits Maintained After Long-Term Telerehabilitation in COPD?. Int J Telerehabil. 2016;8(2):39–48. doi: 10.5195/ijt.2016.6200.

[97] L. Houchen-Wolloff et al., Web-based self-management program (space for copd) for individuals hospitalized with an acute exacerbation of chronic obstructive pulmonary disease: Nonrandomized feasibility trial of acceptability. JMIR Mhealth Uhealth. 2021;9(6). doi: 10.2196/21728.

[98] L. Huniche, B. Dinesen, C. Nielsen, O. Grann, and E. Toft. Patients’ use of self-monitored readings for managing everyday life with COPD: A qualitative study. Telem E Health. 2012;19(5):396–402. doi: 10.1089/tmj.2012.0135.

[99] M. Kargiannakis, D. A. Fitzsimmons, C. L. Bentley, and G. A. Mountain.Does telehealth monitoring identify exacerbations of chronic obstructive pulmonary disease and reduce hospitalisations? an analysis of system data. JMIR Med Inform. 2017;5(1). doi: 10.2196/medinform.6359.

[100] L. Kaye, R. Gondalia, A. Thompson, D. A. Stempel, and M. A. Barrett. The relationship between objective app engagement and medication adherence in asthma and COPD: a retrospective analysis. Sci Rep. 2021;11(1). doi: 10.1038/s41598-021-03827-2.

[101] A. Kjellsdotter, S. Andersson, and M. Berglund. Together for the future – development of a digital website to support chronic obstructive pulmonary disease self-management: A qualitative study. J Multidiscip Healthc. 2021;14:757–766. doi: 10.2147/JMDH.S302013.

[102] L. Knox et al., Assessing the uptake, engagement, and safety of a self-management app, COPD.Pal®, for Chronic Obstructive Pulmonary Disease: a pilot study. Health Technol (Berl). 2021;11(3):557–562. doi: 10.1007/s12553-021-00534-w.

[103] P. B. Koff et al., Impact of proactive integrated care on chronic obstructive pulmonary disease. Chronic Obstr Pulm Dis. 2021;8(1):100-16. doi: 10.15326/JCOPDF.2020.0139.

[104] L. Kooij, P. J. E. Vos, A. Dijkstra, and W. H. van Harten. Effectiveness of a mobile health and self-management app for high-risk patients with chronic obstructive pulmonary disease in daily clinical practice: Mixed methods evaluation study. JMIR Mhealth Uhealth. 2021;9(2). doi: 10.2196/21977.

[105] A. C. K. Lee, S. Oliver, K. Fletcher, and J. Robinson. The impact of telehealth support for patients with diabetes or chronic obstructive pulmonary disease on unscheduled secondary care utilisation: A service evaluation. Inform Prim Care. 2012;20(4):263–270. doi: 10.14236/jhi.v20i4.16.

[106] P. H. Lilholt, L. K. E. Hæsum, and O. K. Hejlesen.Exploring User Experience of a Telehealth System for the Danish TeleCare North Trial,” in Studies in Health Technology and Informatics. IOS Press. 2015:301–305. doi: 10.3233/978-1-61499-512-8-301.

[107] I. Maathuis, V. Margaret Jones, N. Oudshoorn, and I. Maathuis. Telecare and self-management: A guideline for anticipating future care in scenario-based design MobiGuide View project MyoTel View project Telecare and self-management: a guideline for anticipating future care in scenario-based design. IXD&A. 2014;23:142-156. doi: 10.55612/s-5002-023-009.

[108] D. D. Mark, C. Ikehara, C. Matsuura, K. Hara, and D. Li. Validating the impact of teaching pursed-lips breathing with Skype: A pilot study. JHPN. 2013;15(8):424–432, 2013, doi: 10.1097/NJH.0000000000000015.

[109] N. Marquis, P. Larivée, M.-F. Dubois, and M. Tousignant. Are Improvements Maintained After In-home Pulmonary Telerehabilitation for Patients with Chronic Obstructive Pulmonary Disease?. Int J Telerehabil. 2015:21–30. doi: 10.5195/ijt.2014.6156.

[110] H. Mathar, P. Fastholm, and N. S. Larsen. A qualitative study of televideo consultations for COPD patients. Br J Nurs. 2015;24(4):205–209, 2015. doi: 10.12968/bjon.2025.24.4.205.

[111] S. Mierdel and K. Owen. Telehomecare reduces ER use and hospitalizations at William Osler health system. Stud Health Technol Inform. 2015;209:102–108. doi: 10.3233/978-1-61499-505-0-102.

[112] M. Nield and G. W. S. Hoo. Real-time telehealth for COPD self-management using skype. J COPD. 2012;9(6): 611–619. doi: 10.3109/15412555.2012.708067.

[113] M. North et al., A randomised controlled feasibility trial of E-health application supported care vs usual care after exacerbation of COPD: the RESCUE trial. NPJ Digit Med. 2020;3(1). doi: 10.1038/s41746-020-00347-7.

[114] A. Nyberg, M. Tistad, and K. Wadell. Can the COPD web be used to promote self-management in patients with COPD in swedish primary care: a controlled pragmatic pilot trial with 3 month- and 12 month follow-up. Scand J Prim Health Care. 2019;37(1):69–82. doi: 10.1080/02813432.2019.1569415.

[115] M. W. Orme et al., Findings of the chronic obstructive pulmonary disease-sitting and exacerbations trial (COPD-SEAT) in reducing sedentary time using wearable and mobile technologies with educational support: Randomized controlled feasibility trial. JMIR Mhealth Uhealth. 2018;6(4). doi: 10.2196/mhealth.9398.

[116] N. Patel, K. Kinmond, P. Jones, P. Birks, and M. A. Spiteri. Validation of COPDpredictTM: Unique combination of remote monitoring and exacerbation prediction to support preventative management of COPD exacerbations. Int J Chron Obstruct Pulmon Dis. 2021;16:1887–1899. doi: 10.2147/COPD.S309372.

[117] F. Rassouli, D. Boutellier, J. Duss, S. Huber, and M. H. Brutsche. Digitalizing multidisciplinary pulmonary rehabilitation in COPD with a smartphone application: An international observational pilot study. Int J Chron Obstruct Pulmon Dis. 2018; 13:3831–3836. doi: 10.2147/COPD.S182880.

[118] L. Rixon et al., A RCT of telehealth for COPD patient’s quality of life: the whole system demonstrator evaluation. Clin Respir J. 2017;11(4):459–469. doi: 10.1111/crj.12359.

[119] J. L. Rodriguez Hermosa et al., Compliance and Utility of a Smartphone App for the Detection of Exacerbations in Patients With Chronic Obstructive Pulmonary Disease: Cohort Study. JMIR Mhealth Uhealth. 2020;8(3):e15699. doi: 10.2196/15699.

[120] K. Schnoor et al., A Pharmacy-Based eHealth Intervention Promoting Correct Use of Medication in Patients With Asthma and COPD:Nonrandomized Pre-Post Study. J Med Internet Res. 2022;24(^). doi: 10.2196/32396.

[121] A. Sheridan, A. Jennings, S. Keane, A. Power, and P. Kavanagh. A breath of fresh air’ for tackling chronic disease in Ireland? An evaluation of a self-management support service for people with chronic respiratory diseases. Ir J Med. 2020;189:551-556. doi: 10.1007/s11845-019-02081-w/Published.

[122] F. Sieverink, S. Kelders, A. Braakman-Jansen, and J. Van Gemert-Pijnen. Evaluating the implementation of a personal health record for chronic primary and secondary care: A mixed methods approach. BMC Med Inform Decis Mak. 2019;19(1). doi: 10.1186/s12911-019-0969-7.

[123] V. Stamenova et al., Technology-enabled self-management of chronic obstructive pulmonary disease with or without asynchronous remote monitoring: Randomized controlled trial. J Med Internet Res. 2020;22(7). doi: 10.2196/18598.

[124] A. Steventon, S. Tunkel, I. Blunt, and M. Bardsley. Effect of telephone health coaching (Birmingham OwnHealth) on hospital use and associated costs: Cohort study with matched controls. BMJ (Online). 2013;347(7920,). doi: 10.1136/bmj.f4585.

[125] E. P. W. A. Talboom-Kamp et al., The effect of integration of self-management web platforms on health status in chronic obstructive pulmonary disease management in primary care (e-Vita Study): interrupted time series design. J Med Internet Res. 2017;19(8):e8262, 2017. doi: 10.2196/jmir.8262.

[126] E. P. W. A. Talboom-Kamp et al., High level of integration in integrated disease management leads to higher usage in the e-vita study: self-management of chronic obstructive pulmonary disease with web-based platforms in a parallel cohort design. J Med Internet Res. 2017;19(5). doi: 10.2196/JMIR.7037.

[127] E. P. W. A. Talboom-Kamp, M. S. Holstege, N. H. Chavannes, and M. J. Kasteleyn. Effects of use of an eHealth platform e-Vita for COPD patients on disease specific quality of life domains. Respir Res. 2019;20(1):1–9. Doi: 10.1186/s12931-019-1110-2.

[128] M. Tabak, M. Brusse-Keizer, P. van der Valk, H. Hermens, and M. Vollenbroek-Hutten. A telehealth program for self-management of COPD exacerbations and promotion of an active lifestyle: A pilot randomized controlled trial. Int J Chron Obstruct Pulmon Dis. 2014;9:935–944. doi: 10.2147/COPD.S60179.

[129] S. Ter Stal, J. Sloots, A. Ramlal, H. op den Akker, A. Lenferink, and M. Tabak. An embodied conversational agent in an eHealth self-management intervention for chronic obstructive pulmonary disease and chronic heart failure: Exploratory study in a real-life setting. JMIR Hum Factors. 2021;8(4):e24110, 2021. doi: 10.2196/24110.

[130] A. R. van Buul et al., A systematic diagnostic evaluation combined with an internet-based self-management support system for patients with asthma or COPD. Int J Chron Obstruct Pulmon. Dis. 2018;13:3297–3306. doi: 10.2147/COPD.S175361.

[131] A. R. Van Buul, C. Derksen, O. Hoedemaker, O. Van Dijk, N. H. Chavannes, and M. J. Kasteleyn. eHealth program to reduce hospitalizations due to acute exacerbation of chronic obstructive pulmonary disease: Retrospective study. JMIR Form Res. 2021;5(3). doi: 10.2196/24726.

[132] M. van der Heijden, P. J. F. Lucas, B. Lijnse, Y. F. Heijdra, and T. R. J. Schermer. An autonomous mobile system for the management of COPD. J Biomed Inform. 2013;46(3):458–469. doi: 10.1016/j.jbi.2013.03.003.

[133] F. Van Lieshout et al., Evaluating the implementation of a remote-monitoring program for chronic obstructive pulmonary disease: Qualitative methods from a service design perspective. J Med Internet Res. 2020;22(10). doi: 10.2196/18148.

[134] C. M. van Zelst et al., The impact of the involvement of a healthcare professional on the usage of an eHealth platform: a retrospective observational COPD study. Respir Res. 2021;22(1). doi: 10.1186/s12931-021-01685-0.

[135] T. K. Vatnøy, E. Thygesen, and B. Dale. Telemedicine to support coping resources in home-living patients diagnosed with chronic obstructive pulmonary disease: Patients’ experiences. J Telemed Telecare. 2017;23(1):126–132. doi: 10.1177/1357633X15626854.

[136] S. Vorrink, C. Huisman, H. Kort, T. Troosters, and J. W. Lammers. Perceptions of patients with chronic obstructive pulmonary disease and their physiotherapists regarding the use of an eHealth intervention. JMIR Hum Factors. 2017;4(3). doi: 10.2196/humanfactors.7196.

[137] J. Walters et al., Effects of telephone health mentoring in community-recruited chronic obstructive pulmonary disease on self-management capacity, quality of life and psychological morbidity: A randomised controlled trial. BMJ Open. 2013;3(9). doi: 10.1136/bmjopen-2013-003097.

[138] P. Zanaboni, L. A. Lien, A. Hjalmarsen, and R. Wootton. Long-term telerehabilitation of COPD patients in their homes: Interim results from a pilot study in Northern Norway. J Telemed Telecare. 2013;19(7):425–429. doi: 10.1177/1357633X13506514.

[139] P. Zanaboni, H. Hoaas, L. Aarøen Lien, A. Hjalmarsen, and R. Wootton. Long-term exercise maintenance in COPD via telerehabilitation: a two-year pilot study. J Telemed Telecare. 2017;23(1):74–82. doi: 10.1177/1357633X15625545.
